# Supplementary material for: Best holdout assessment is sufficient for cancer transcriptomic model selection
Source: Patterns (N Y). 2024 Dec 6;5(12):101115. doi: 10.1016/j.patter.2024.101115 (PMC11701843; doi:10.1016/j.patter.2024.101115)
Supplement: Document S1. Figures S1–S10 and Note S1 [file mmc1.pdf]

Patterns, Volume *5*

Supplemental information

Best holdout assessment is sufficient for cancer transcriptomic model selection

Jake Crawford, Maria Chikina, and Casey S. Greene

Supplementary Material

Supplementary Note S1

We were interested in exploring the extent to which excluding the target gene's expression profile from the input features affects performance, if at all. Additionally, since our labels include both point mutations and copy number changes, we sought to determine whether the answer to this question depends on the inclusion of copy number changes in the label set for a particular gene. To test this across driver genes, we calculated the contribution of single nucleotide variant (SNV) and copy number variant (CNV) changes to each gene's positively labeled sample set, and picked ten genes where CNV changes make up a relatively large proportion of positive labels, and ten genes where CNV changes make up a small proportion of positive labels. Genes where positive labels commonly result from CNV changes are as follows:

Gene	SNV sample count	SNV + CNV count	SNV / (SNV + CNV) ratio
BAP1	105	146	0.719
CDKN2A	288	1308	0.220
EGFR	192	444	0.432
ERBB2	129	440	0.293
GNAS	99	266	0.372
KDM6A	163	295	0.553
PDGFRA	131	235	0.557
PTEN	584	985	0.593
RB1	259	522	0.496
SMAD4	131	289	0.453

And genes where samples are rarely positively labeled based on CNV changes:

Gene	SNV sample count	SNV + CNV count	SNV / (SNV + CNV) ratio
ARID1A	588	629	0.934
ATRX	455	508	0.896
BRAF	569	605	0.940
CTNNB1	297	304	0.977
EP300	256	265	0.966
IDH1	414	415	0.997
NRAS	169	170	0.994
RNF43	152	157	0.968
SETD2	252	279	0.903
TP53	3305	3372	0.980

We also considered baseline model performance in the choice of these gene sets. If a gene has a very low or very high SNV / (SNV + CNV) ratio but the associated classifier generally performs poorly, we wouldn't expect to observe a performance change, regardless of the input features. For this experiment, the 20 genes we selected all had a reasonably high performance baseline, to maximize our ability to observe changes if they occur.

We visualized the mean difference in performance for the best-performing and "smallest good" models (LASSO parameters) with the "control" set of features, as compared to the best-performing and "smallest good" models with the "drop target" set of features (all of the gene expression features except the target gene), shown in Figure S1. In general, we do observe that performance tends to be better for the "control" models, although there are some exceptions (EGFR, ERBB2, PDGFRA, PTEN, EP300) where the "drop target" model actually performs slightly better. We do observe that there are some genes (BAP1, CDKN2A, KDM6A, RB1, ARID1A, ATRX) where performance decreases considerably when the target gene is not present in the feature set. For both the "best" and "smallest good" model selection approaches, this effect is slightly more consistent in the "frequent CNV" gene set than in the "rare CNV" gene set (mean control - drop target difference of 0.021/0.019 in the "frequent CNV" genes as compared to 0.009/0.004 in the "rare CNV" genes), but in both cases there is considerable variance between genes.

Based on these results, given the observation that the mean difference in model performance is fairly small in both "frequent CNV" and "rare CNV" cases, and for both model selection approaches, we conclude that combining point mutation and CNV data and including the target gene in the feature set are reasonable general rules for our pan-cancer and pan-gene study. In general, our focus is less on individual prediction performance and more on model complexity, which is another degree removed from the individual prediction performance. In addition, including the target gene would seem most likely to increase the benefit of smaller models, as the single-gene could be considered particularly information rich. While these results don't seem to heavily influence our experiment examining generality, the exceptions we noted above emphasize the importance of considering the biological context in applications to specific driver genes or prediction problems.

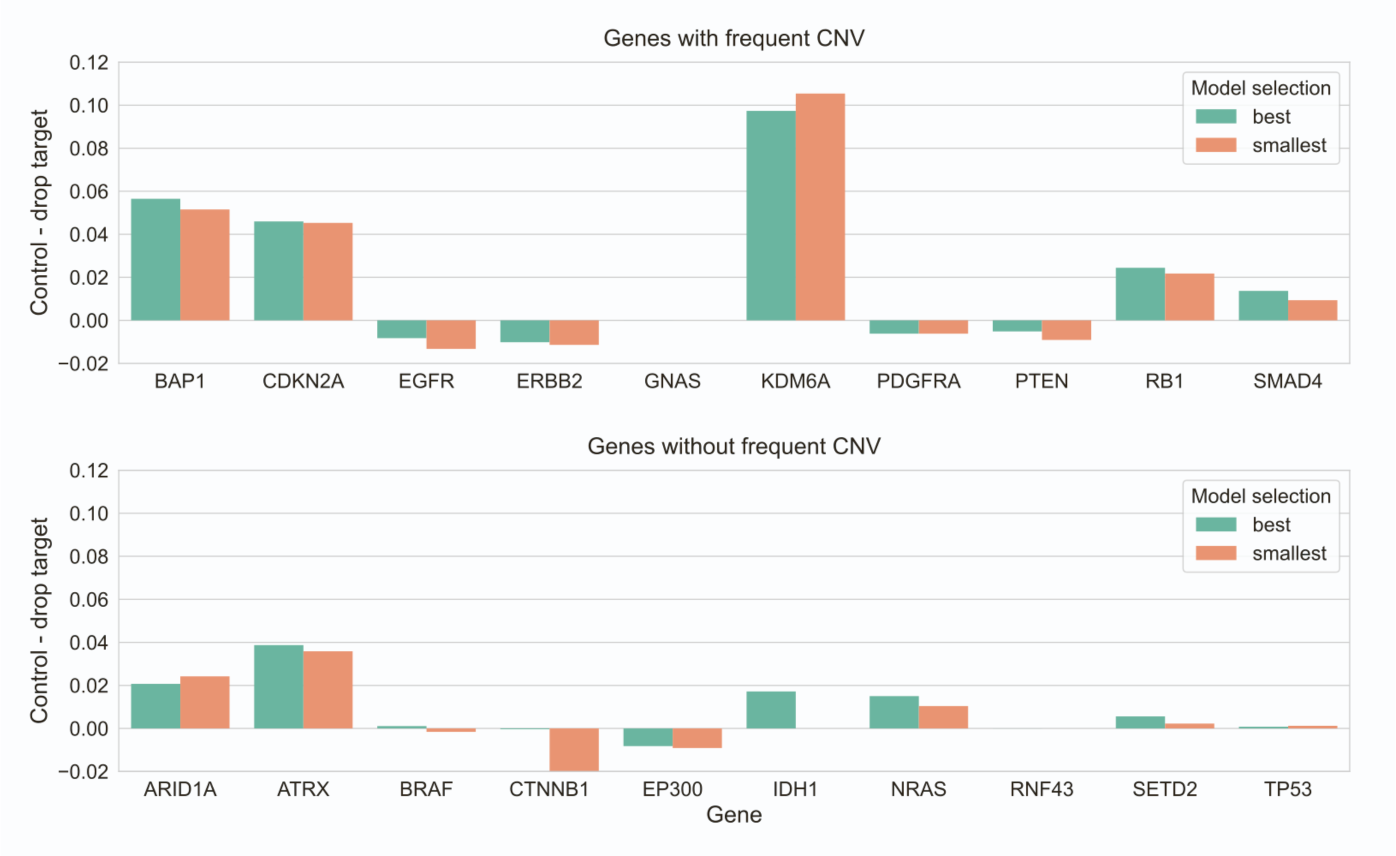

Figure S1: Bar plot showing difference in performance (AUPR) between models including and excluding the target gene, for genes where CNV changes are (top) and are not (bottom) frequently included in the label set, colored by model selection approach. Positive values represent better performance for the “control” model, and negative values better performance for the “drop target” model.

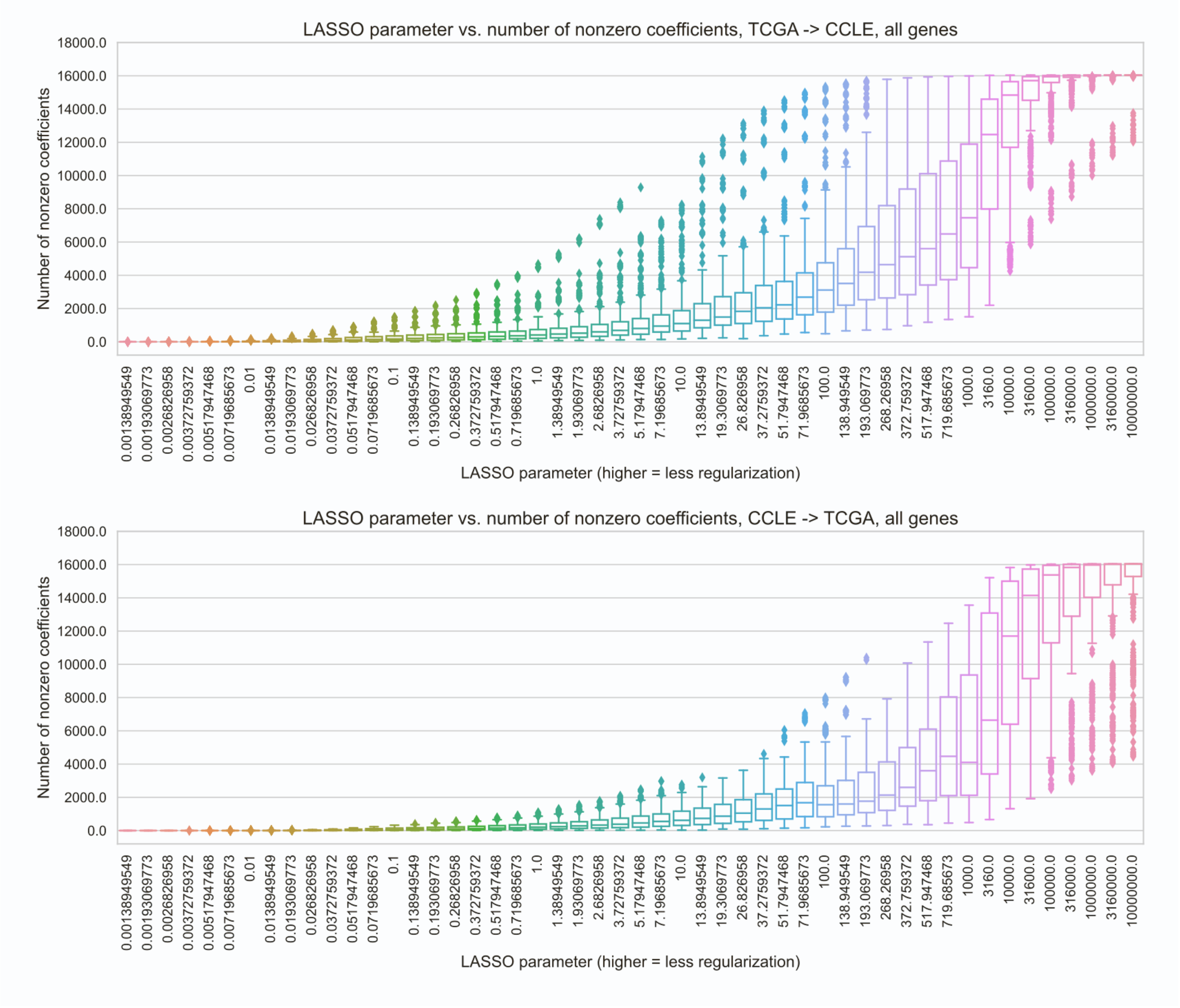

Figure S2: Number of nonzero coefficients (model sparsity) across varying regularization parameters, for 71 genes (TCGA to CCLE prediction, top) and 70 genes (CCLE to TCGA prediction, bottom) in the Vogelstein et al. dataset.

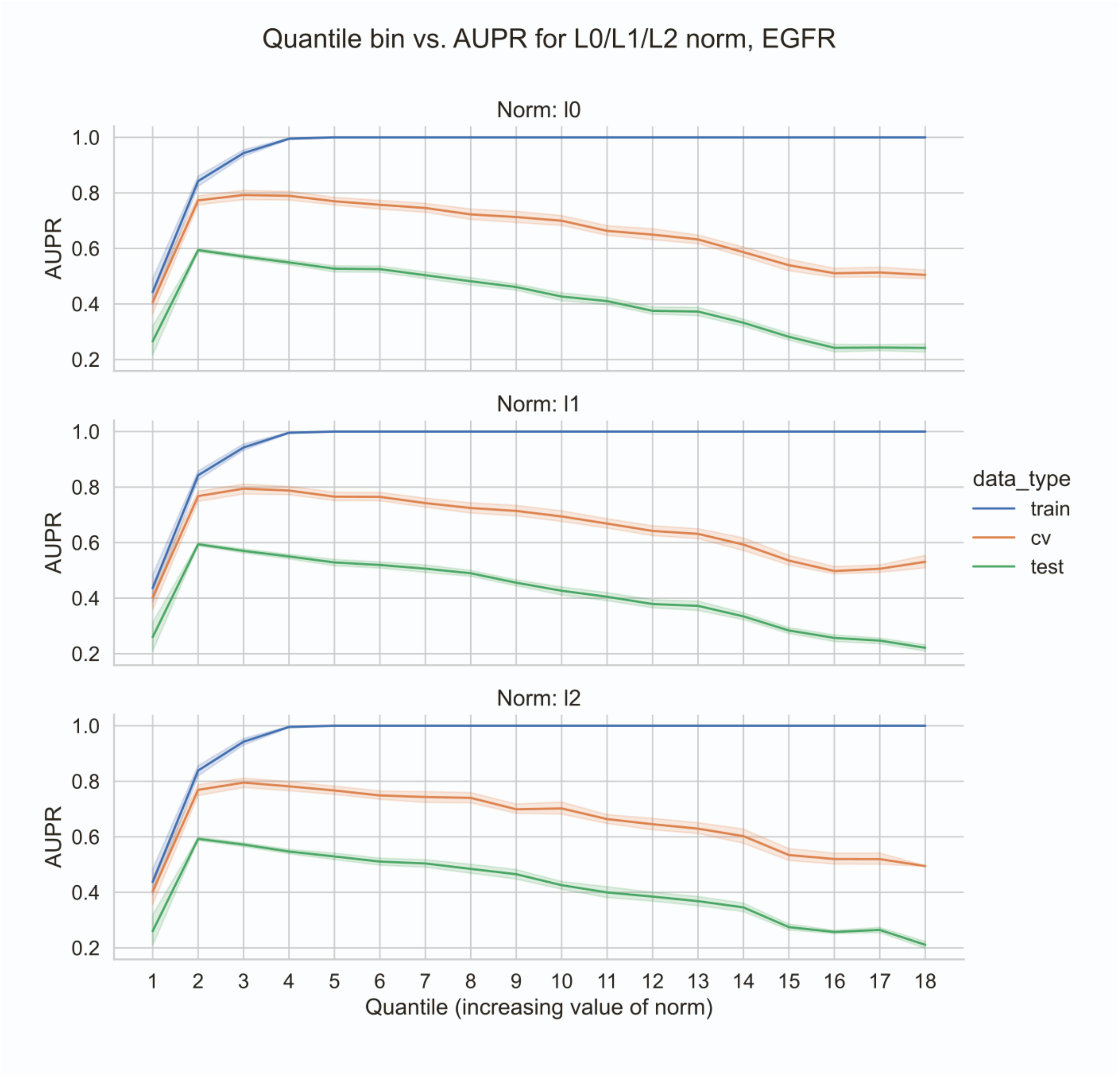

Figure S3: Value of norm of coefficient vector vs. performance, for EGFR mutation status prediction from TCGA to CCLE. The x-axis shows the value of each norm for each model, binned into quantiles in order to plot results on the same axis since each norm has a different scale.

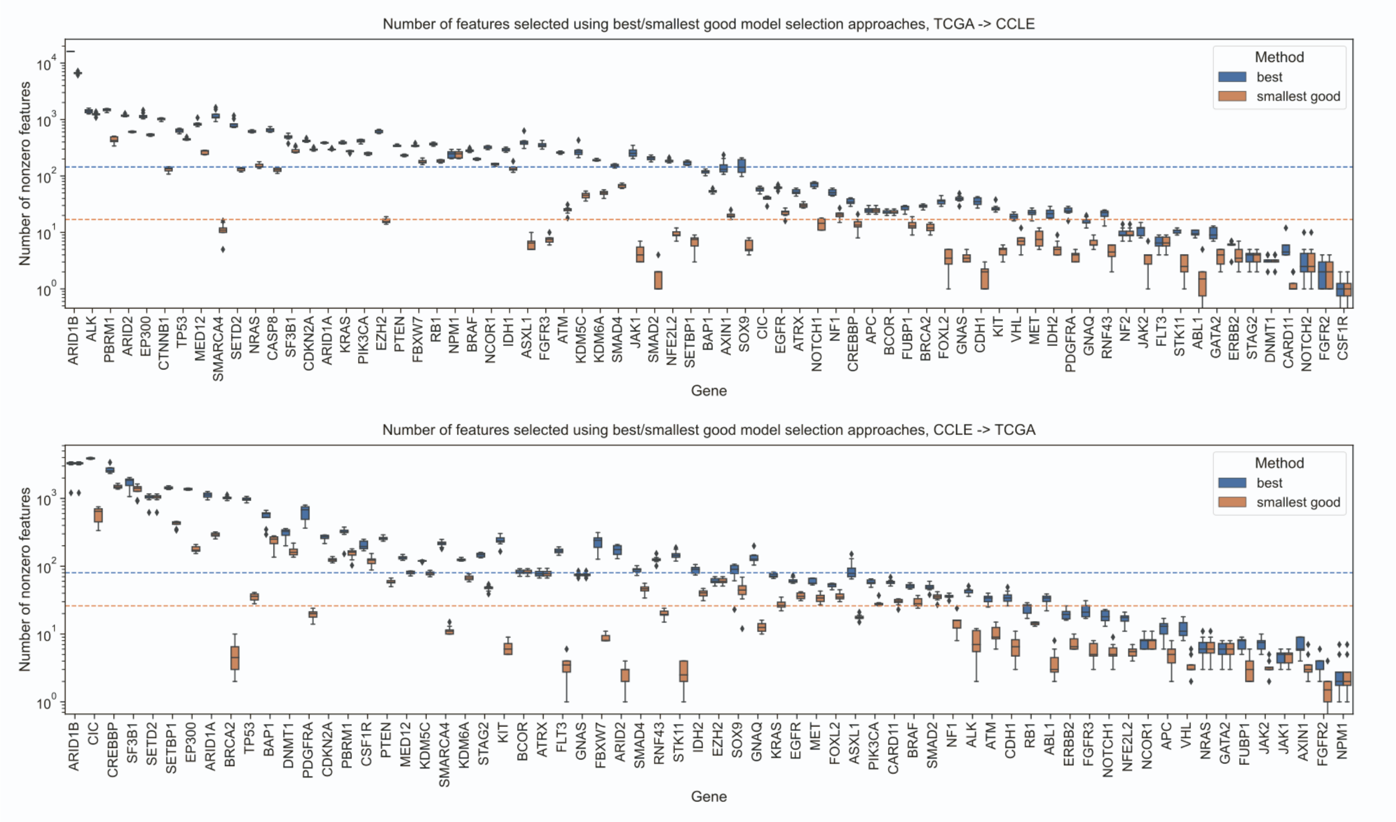

Figure S4: Distributions of number of features selected by the “best” and “smallest good” models, across seeds and folds, for TCGA to CCLE (top) and CCLE to TCGA (bottom) mutation prediction. Dotted lines show the median number of features for the best (blue) and smallest good (orange) numbers across genes: TCGA to CCLE - median of 144 features for the “best” approach and 17 features for the “smallest good” approach; CCLE to TCGA - median of 80 features for the “best” approach and 26 features for the “smallest good” approach.

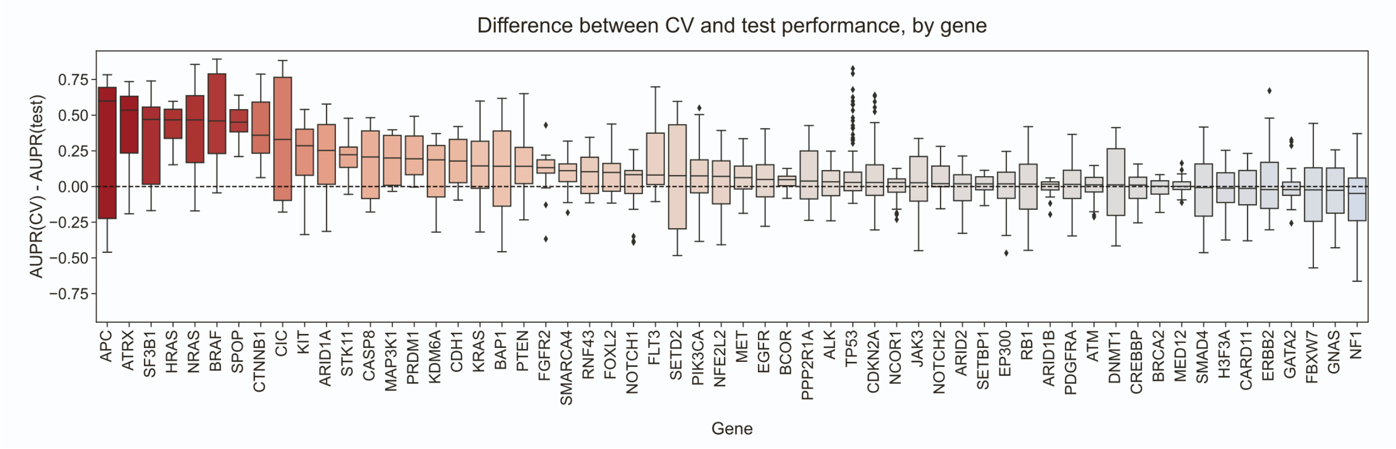

Figure S5: Distributions of performance difference between cross-validation data (same cancer types as training data) and holdout data (cancer types not represented in data), grouped by held-out gene. Each point shows performance for a single train/validation split for one cancer type that was held out, using a classifier trained to predict mutations in the given gene.

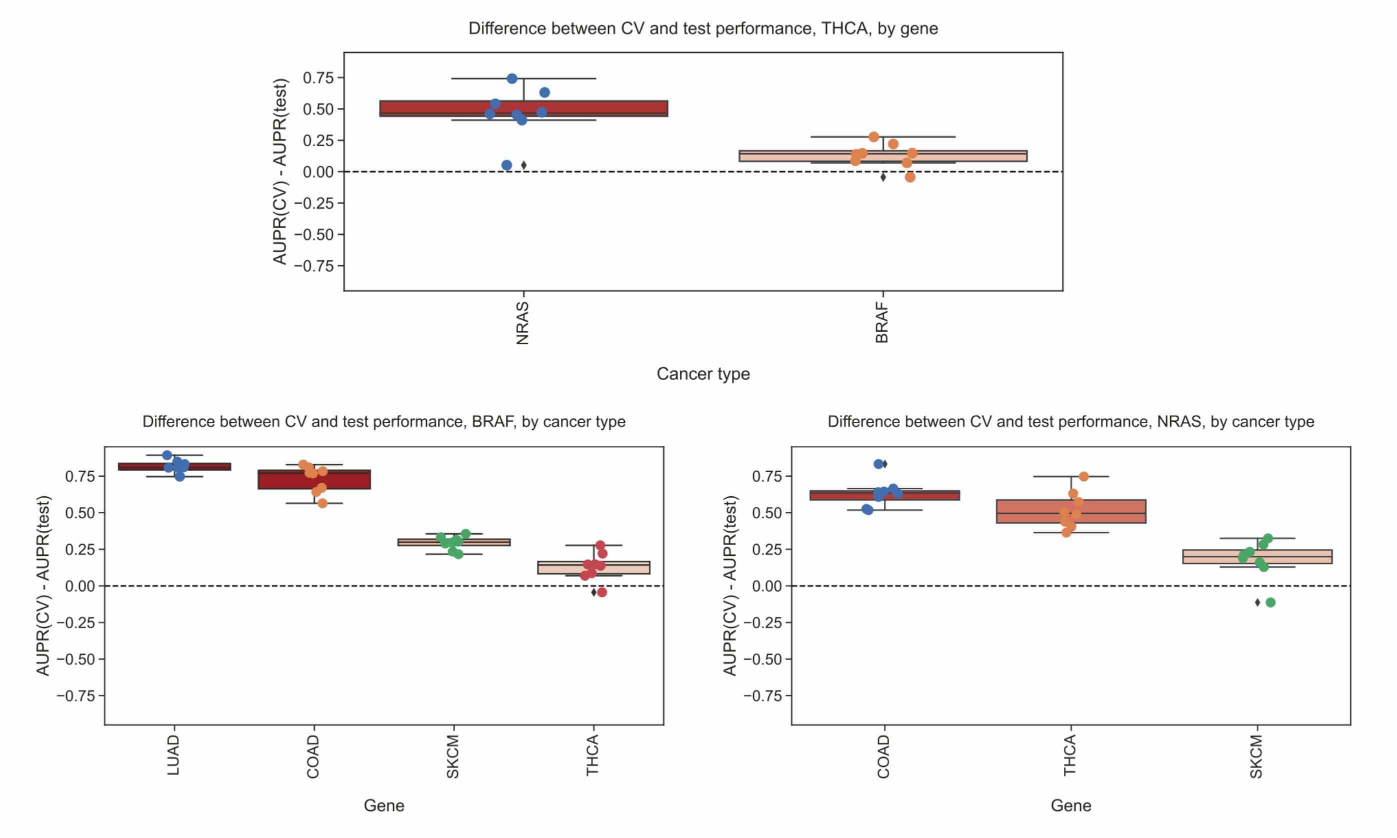

Figure S6: Top row: Distribution of performance differences when thyroid cancer (THCA) data is held out from training set across seeds/folds, grouped by gene. Bottom row: Distributions of performance differences for genes where THCA is included in training/holdout sets, relative to other cancer types that are included.

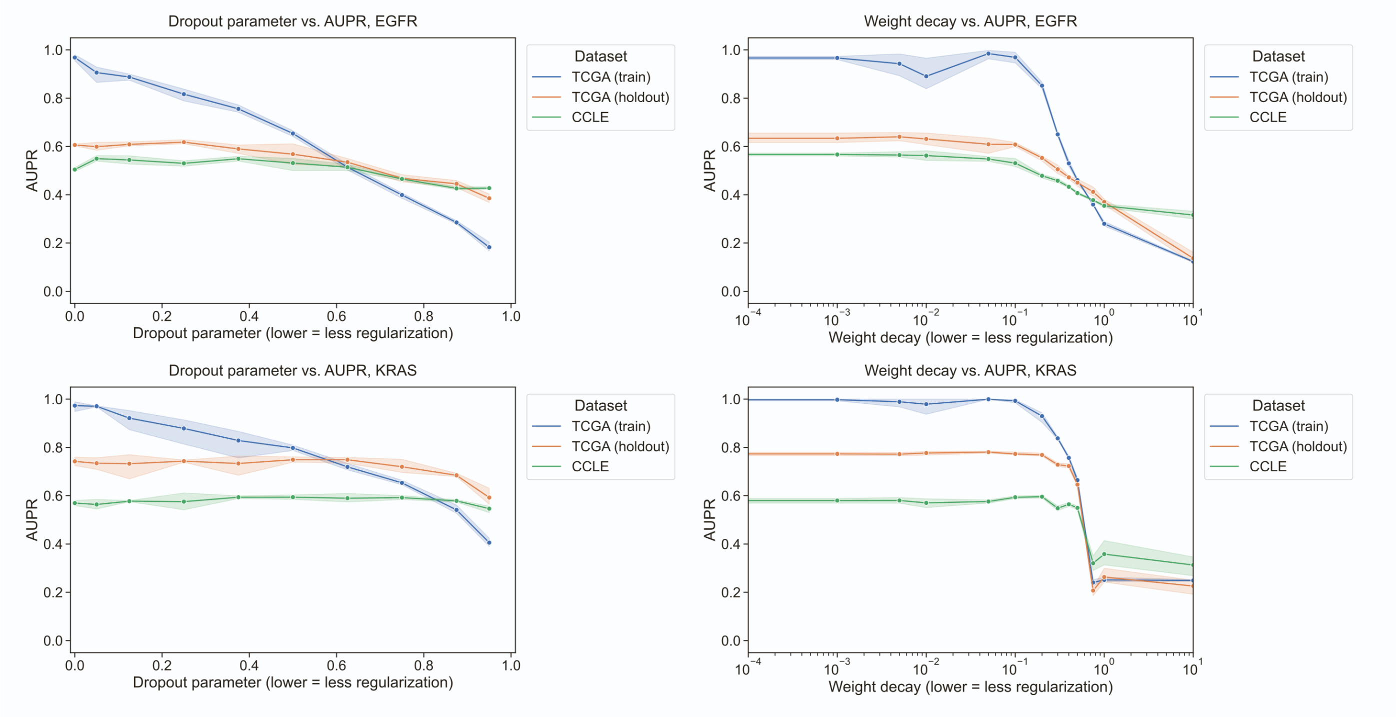

Figure S7: Performance vs. dropout parameter (first column) and weight decay strength (second column), for EGFR mutation prediction (first row) and KRAS mutation prediction (second row) using a 3-layer fully connected neural network trained on TCGA (blue/orange) and evaluated on CCLE (green).

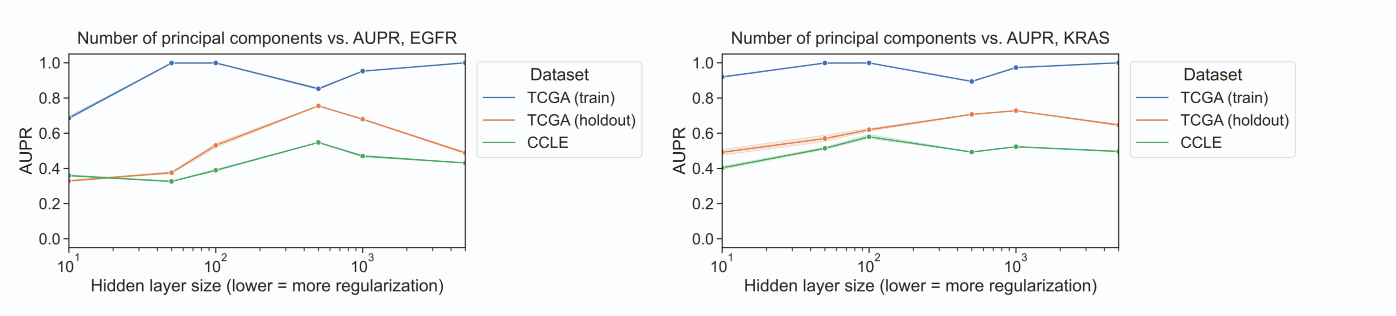

Figure S8: Performance vs. number of gene expression principal components, used as input to a 3-layer fully connected neural network trained on TCGA (blue/orange) and evaluated on CCLE (green), for EGFR and KRAS mutation status prediction.

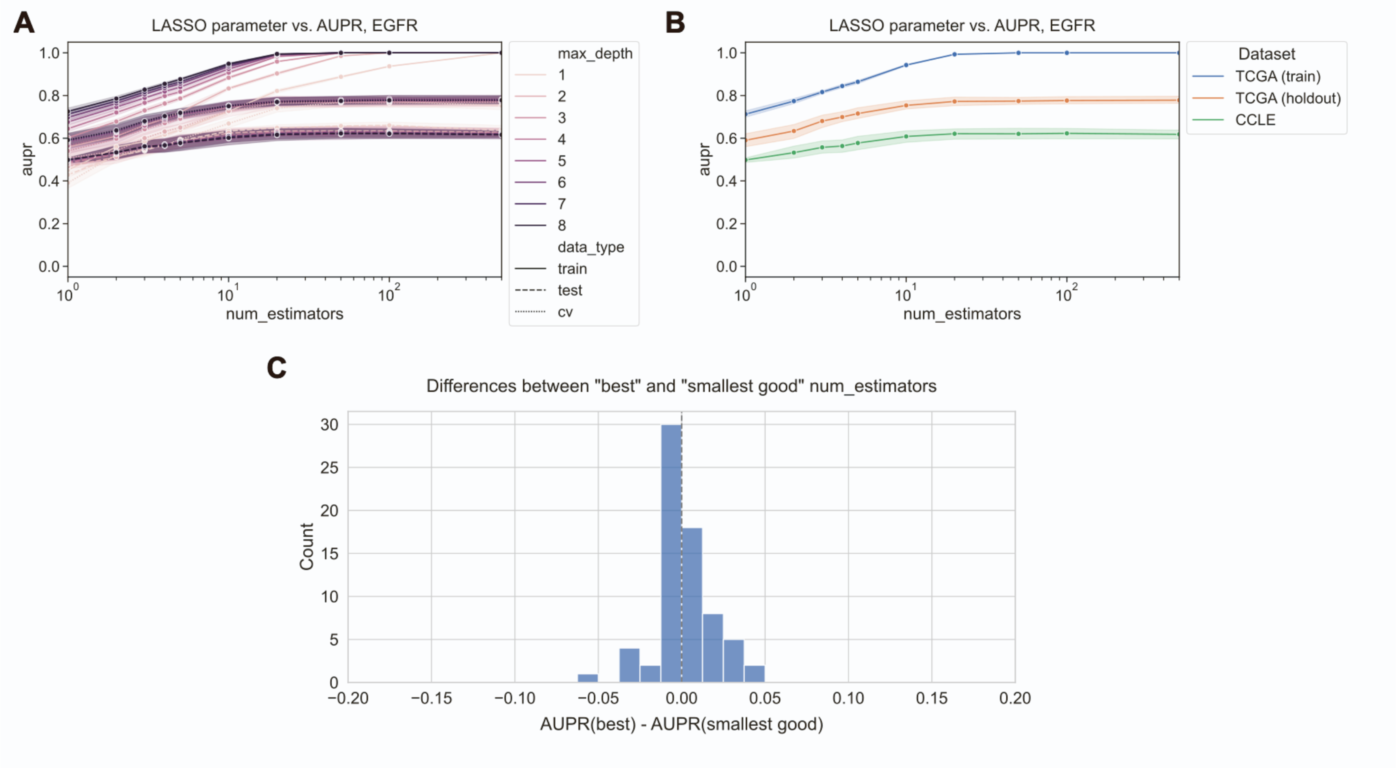

Figure S9: Performance across regularization parameter values for XGBoost mutation status classification, for generalization from TCGA to CCLE. Top row shows performance for EGFR across varying values of `num_estimators` and `max_depth` (Panel A), and for `max_depth=8` across a range of `num_estimators` (Panel B). Panel C summarizes the distribution of performance comparisons between "best" vs. "smallest good" `num_estimators` (33/71 genes best > smallest good, 17/71 smallest good > best, 20/71 best = smallest good).

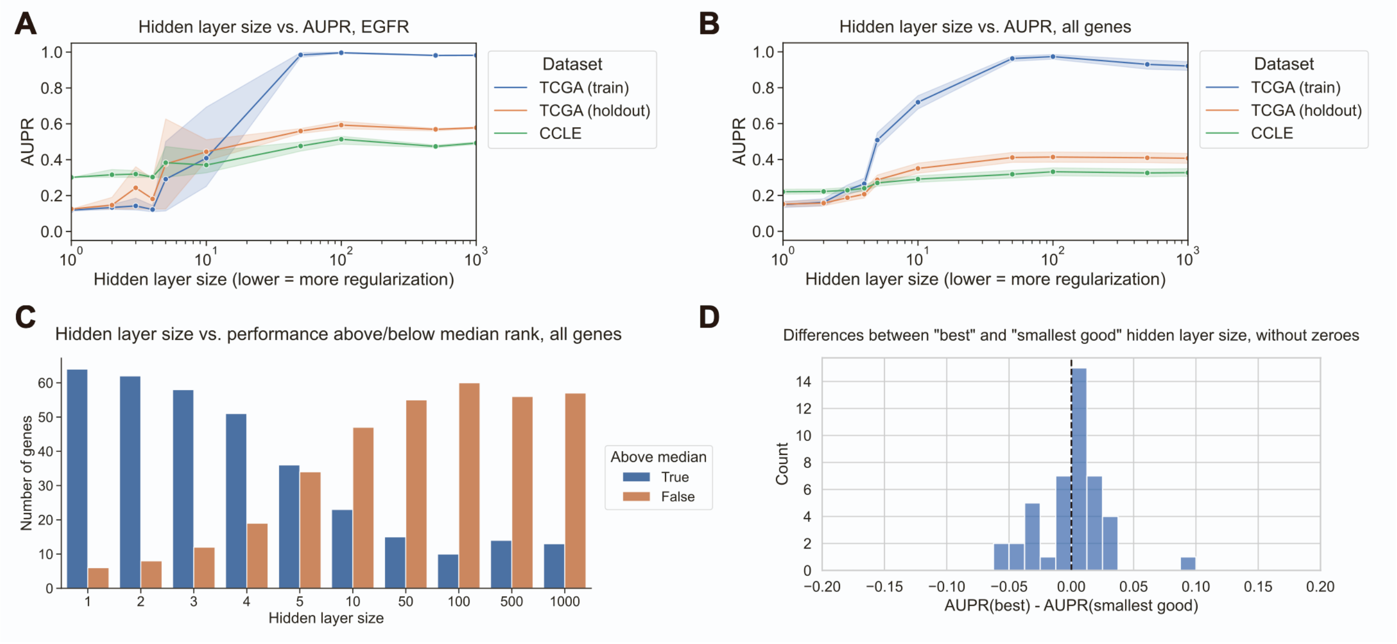

Figure S10: Summary of performance for TCGA to CCLE generalization using 5-layer fully connected neural network, analogous to results shown in Figure 5 for 3-layer network. All experiments used expression of top 8000 genes by mean absolute deviation, for computational reasons. In the "best" vs. "smallest good" analysis, 27/71 genes had better performance for the best model, and 17/71 had better performance for the smallest good model, with 26/71 genes where the best and smallest good models were equal.
