# Supplementary material for: Best holdout assessment is sufficient for cancer transcriptomic model selection
Source: Patterns (N Y). 2024 Dec 6;5(12):101115. doi: 10.1016/j.patter.2024.101115 (PMC11701843; doi:10.1016/j.patter.2024.101115)
Supplement: Document S2. Article plus supplemental information [file mmc2.pdf]

Patterns

Article

Best holdout assessment is sufficient for cancer transcriptomic model selection

Highlights

- Systematic evaluation of generalization of cancer transcriptomics predictive models
- Cross-validation performance is equally indicative as model size/complexity
- Similar results hold broadly across generalization contexts and model types

Authors

Jake Crawford, Maria Chikina, Casey S. Greene

Correspondence

casey.s.greene@cuanschutz.edu

In brief

Simpler models can have advantages over more complex ones, including cost, interpretability, and, some have suggested, improved generalization across datasets or biological contexts. Here, the authors directly test the latter assumption in the context of gene signatures and find that smaller signatures do not consistently generalize better. The authors recommend choosing models that perform best on held-out data or in cross-validation instead of those that are smaller or more regularized.

Crawford et al., 2024, Patterns *5*, 101115
December 13, 2024 © 2024 The Authors. Published by Elsevier Inc.
https://doi.org/10.1016/j.patter.2024.101115

CellPress

Patterns

CellPress
OPEN ACCESS

Article

Best holdout assessment is sufficient for cancer transcriptomic model selection

Jake Crawford,[1] Maria Chikina,[2] and Casey S. Greene[3,4,5,*]
[1]Genomics and Computational Biology Graduate Group, Perelman School of Medicine, University of Pennsylvania, Philadelphia, PA, USA
[2]Department of Computational and Systems Biology, School of Medicine, University of Pittsburgh, Pittsburgh, PA, USA
[3]Department of Biomedical Informatics, University of Colorado School of Medicine, Aurora, CO, USA
[4]Center for Health AI, University of Colorado School of Medicine, Aurora, CO, USA
[5]Lead contact
*Correspondence: casey.s.greene@cuanschutz.edu
https://doi.org/10.1016/j.patter.2024.101115

THE BIGGER PICTURE Existing recommendations in statistics and machine learning suggest that smaller, or simpler, predictive models are more likely to generalize well. In cancer transcriptomics, this manifests as a preference for small "gene signatures," or groups of genes whose expression is used to define subtypes or suggest therapeutic interventions. This study uses public datasets to test the generalization performance of cancer gene expression-based predictive models both across datasets (from cell lines to tumor samples and vice versa) and across cancer types/tissues of origin. In general, we do not observe strong evidence that simpler models inherently generalize more effectively than more complex ones. Our results underscore the importance of defining clear goals in machine learning-based transcriptomic analyses. If the goal is to achieve robust performance across contexts or datasets, then we recommend directly evaluating generalization whenever possible; otherwise, we recommend choosing the model that performs the best on unseen data via cross-validation.

SUMMARY

Guidelines in statistical modeling for genomics hold that simpler models have advantages over more complex ones. Potential advantages include cost, interpretability, and improved generalization across datasets or biological contexts. We directly tested the assumption that small gene signatures generalize better by examining the generalization of mutation status prediction models across datasets (from cell lines to human tumors and vice versa) and biological contexts (holding out entire cancer types from pan-cancer data). We compared model selection between solely cross-validation performance and combining cross-validation performance with regularization strength. We did not observe that more regularized signatures generalized better. This result held across both generalization problems and for both linear models (LASSO logistic regression) and non-linear ones (neural networks). When the goal of an analysis is to produce generalizable predictive models, we recommend choosing the ones that perform best on held-out data or in cross-validation instead of those that are smaller or more regularized.

INTRODUCTION

Gene expression datasets are typically "wide," with many gene features and relatively few samples. These feature-rich datasets present obstacles in many aspects of machine learning, including overfitting and multicollinearity, and challenges in interpretation. To facilitate the use of feature-rich gene expression data in machine learning models, feature selection and/or dimension reduction are commonly used to distill a more condensed data representation from the input space of all genes.[1,2] The intuition is that many gene expression features are likely irrelevant to the prediction problem, redundant, or contain no meaningful variation across samples, so transforming them or selecting a subset can generate a more reliable predictor.

In cancer transcriptomics, this preference for small, parsimonious sets of genes can be seen in the popularity of "gene signatures." These are groups of genes whose expression levels are used to define cancer subtypes or predict prognosis or therapeutic response.[3,4] Many studies specify the size of the signature in the paper's title or abstract, suggesting that the fewer genes in a gene signature, the better, e.g., Chen et al.,[5] Landemaine et al.,[6] and Cardoso et al.[7] Clinically, there are many

© 2024 The Authors. Published by Elsevier Inc.
This is an open access article under the CC BY license (http://creativecommons.org/licenses/by/4.0/).

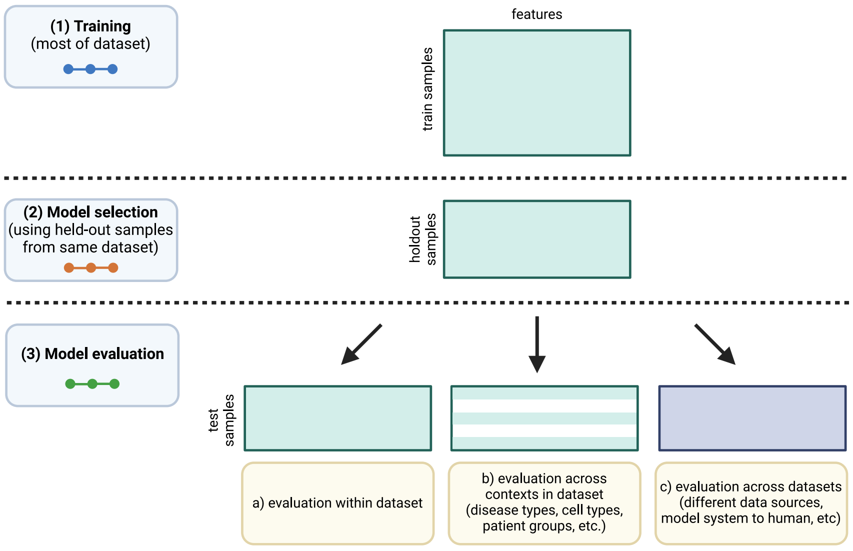

Figure 1. Schematic of experimental design

In the regularization vs. performance curves in Figures 2, 3, 4, and 5, colors correspond to components of model evaluation shown in the schematic (i.e., blue for training data performance, orange for holdout performance on the same dataset, and green for generalization performance across datasets or biological contexts).

reasons why a smaller gene signature may be preferable, including cost (fewer genes may be less expensive to profile or validate, whereas a large signature likely requires a targeted array or next-generation sequencing [NGS] analysis[8]) and interpretability (it is easier to reason about the function and biological role of a smaller gene set than a large one since even disjoint gene signatures tend to converge on common biological pathways[9,10]).

Behind much of this work, there is an underlying assumption that smaller gene signatures tend to be more robust: that for a new patient or in a new biological context, a smaller gene set or more parsimonious model will be more likely to maintain its predictive performance than a larger one. Similar ideas are described in the statistics literature, suggesting that simpler models with performances that are comparable to the best model are more likely to perform robustly across datasets or resist overfitting.[11,12] Although these assumptions have rarely been formally stated or systematically tested in genomics applications, they are often included in guidelines or rules of thumb for applied statistical modeling or machine learning in biology, e.g., Altman and Royston,[13] Boulesteix et al.,[14] and Kass et al.[15]

In this study, we sought to test the robustness assumption directly by evaluating model generalization across biological contexts, inspired by previous work on domain adaptation and transfer learning in cancer transcriptomics.[16–18] We used two large, heterogeneous public cancer datasets: The Cancer Genome Atlas (TCGA) for human tumor sample data[19] and the Cancer Cell Line Encyclopedia (CCLE) for human cell line data.[20] These datasets contain overlapping -omics data types derived from distinct data sources, allowing us to quantify model generalization across data sources. In addition, each dataset contains samples from a wide range of different cancer types/tissues of origin, allowing us to quantify model generalization across cancer types. We trained both linear and non-linear models to predict mutation status (presence or absence) from RNA sequencing (RNA-seq) gene expression for approximately 70 cancer driver genes across varying levels of model simplicity and degrees of regularization, resulting in a variety of gene signature sizes. We compared two simple procedures for model selection, one that combines cross-validation performance with model parsimony and one that only relies on cross-validation performance, for each classifier in each context.

Our results suggest that, in general, mutation status classification models that perform well in cross-validation within a biological context also generalize well across biological contexts. There are some individual genes and some individual cancer types where more regularized, well-performing models outperform the best-performing model. However, we do not observe a systematic generalization advantage for smaller/more regularized models across all genes and cancer types. These results provide evidence that good cross-validation performance within a biological context (data source or cancer type) is a sufficient proxy for robust performance across contexts.

RESULTS

Evaluating model generalization using public cancer data

We collected data from TCGA Pan-Cancer Atlas and CCLE to predict the presence or absence of mutations in cancer genes as a benchmark of cancer-related information content across cancer types and contexts. We trained mutation status classifiers across approximately 70 genes involved in cancer development and progression from Vogelstein et al.[21] using LASSO logistic regression with gene expression (RNA-seq) values as predictive features and integrating point mutation and copy-number data to label each sample as mutated or not mutated in the target gene (Note S1). We fit each classifier across a variety of regularization parameters, resulting in models with a variety of different sparsity levels between the extremes of 0 nonzero features and all features included (Figure S2). Inspired by the generalization experiments across tissues and model systems in Ma et al.,[16] we designed experiments to evaluate the generalization of mutation status classifiers across datasets (TCGA to CCLE and CCLE to TCGA) and across biological contexts (cancer types) within TCGA, relative to a within-dataset baseline (Figure 1).

Generalization from human tumor samples to cell lines is more effective than the reverse

To evaluate "cross-dataset" generalization, we trained mutation status classifiers on human tumor data from TCGA and evaluated them on cell line data from CCLE, as well as the reverse, from the CCLE to TCGA. As an example, we examined *EGFR*,

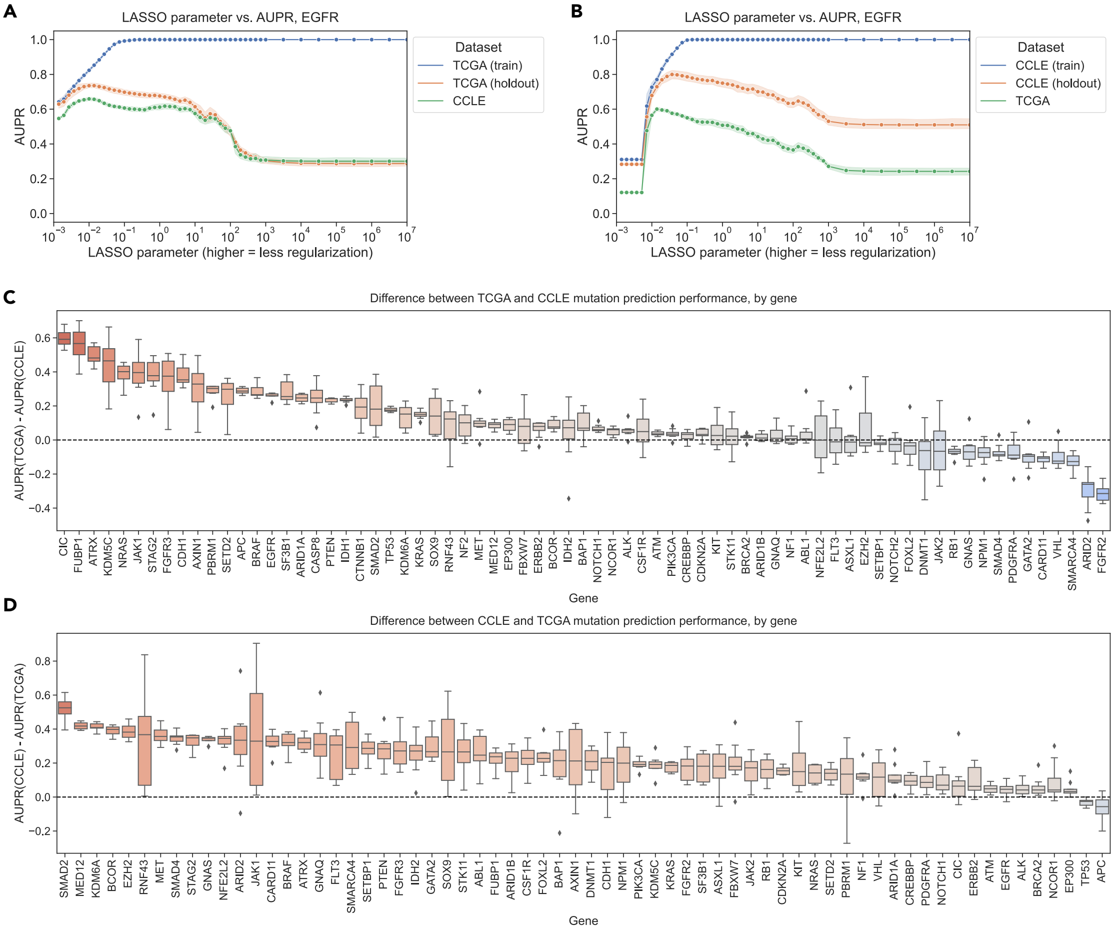

Figure 2. Evaluating generalization across cell lines and tumor samples

(A) *EGFR* mutation status prediction performance on training samples from TCGA (blue), held-out TCGA samples (orange), and CCLE samples (green) across varying LASSO parameters. Shading represents 95% confidence intervals derived from 1,000 bootstrap samples at each tested level of regularization.

(B) *EGFR* mutation status prediction performance on training samples from CCLE (blue), held-out CCLE samples (orange), and TCGA samples (green).

(C) Difference in mutation status prediction performance for models trained on TCGA (holdout data) and evaluated on CCLE (test data), after correcting for baseline mutation frequency, across 71 genes from Vogelstein et al. For each gene, the best model (LASSO parameter) was selected using holdout AUPR performance. Genes on the x axis are ordered by median AUPR difference across cross-validation splits from highest to lowest.

(D) Difference in mutation status prediction performance for models trained on CCLE (holdout data) and evaluated on TCGA (test data) across 66 genes from Vogelstein et al.

an oncogenic tyrosine kinase that is commonly mutated in diverse cancer types and cancer cell lines, including lung cancer, colorectal cancer, and glioblastoma.[22,23] For *EGFR* mutation status classifiers trained on TCGA and evaluated on CCLE, we saw that the area under the precision-recall curve (AUPR) on cell lines was slightly worse than on held-out tumor samples but comparable across regularization levels/LASSO parameters (Figure 2A). On the other hand, *EGFR* classifiers trained on CCLE and evaluated on TCGA performed considerably worse on human tumor samples as compared to held-out cell lines (Figure 2B). When we compared performance with norms of model coefficient vectors, including the L_1 norm that LASSO models explicitly optimize, as opposed to the LASSO parameter values, the observed performance trends were similar (Figure S3).

To explore these tendencies more generally, we compared performance across all genes in the Vogelstein et al. dataset for both TCGA-to-CCLE and CCLE-to-TCGA generalization. We measured the difference between performance on the holdout data within the training dataset and performance across datasets after correcting for the baseline frequency of mutation occurrence in the relevant dataset (i.e., the expected AUPR value for a random classifier). A positive difference indicates poor generalization (better holdout performance than test performance), and a 0 or negative difference indicates good generalization (comparable test performance to holdout performance). For generalization from TCGA to CCLE, we observed that median AUPR differences were mostly centered around 0 for most genes, with some exceptions at the extremes (Figure 2C; performance differences on the y axis). An example of a gene exhibiting poor generalization was *IDH1*, shown toward the left of Figure 2C as having good performance on held-out TCGA data and poor performance on CCLE data. IDH-mutant glioma cell lines are poorly represented compared to IDH-mutant patient tumors, which may explain the difficulty of generalization to cell

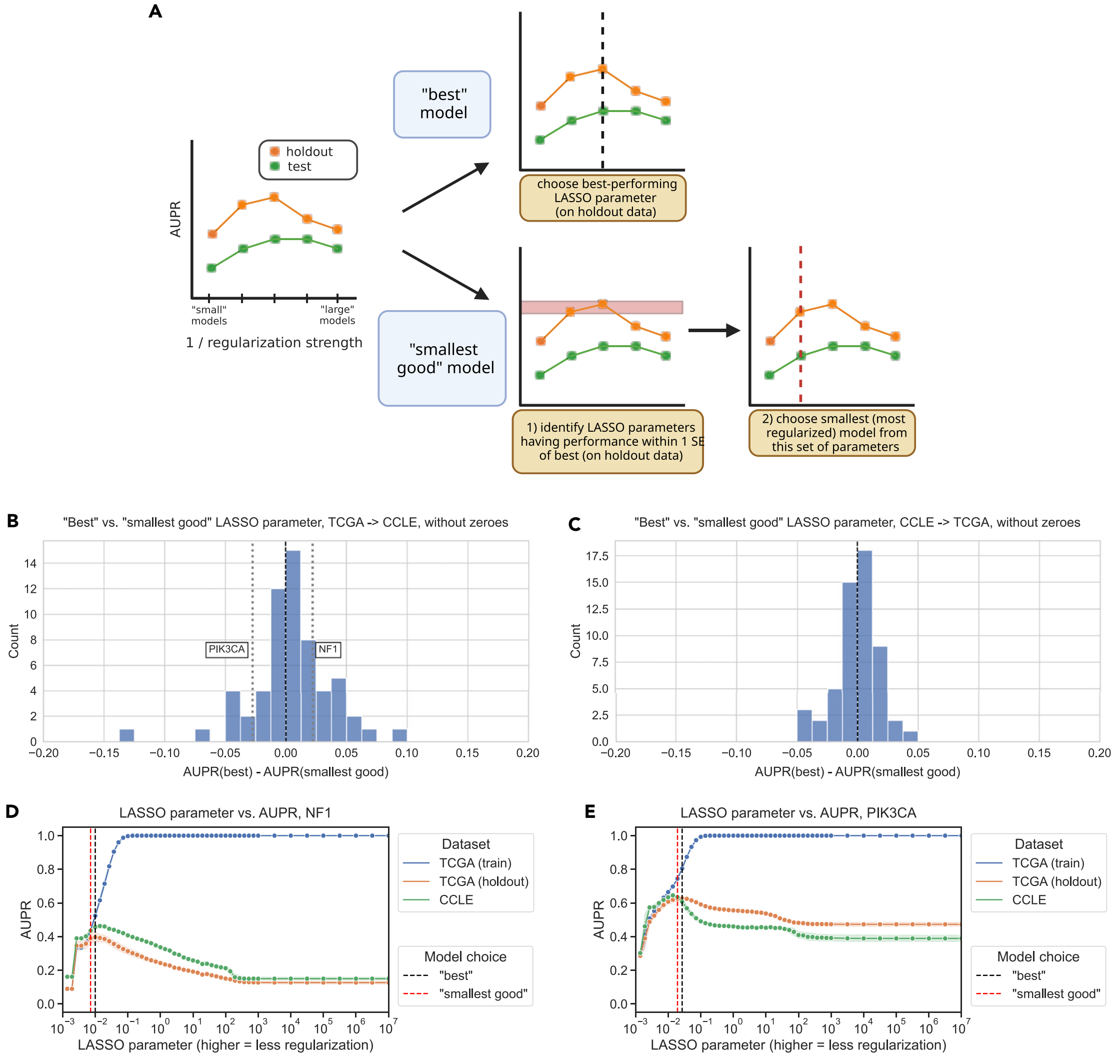

Figure 3. Quantifying and comparing models across the spectrum of size/complexity

(A) Schematic of best vs. smallest good model comparison experiments.

(B) Distribution of performance comparisons between best and smallest good model selection strategies for TCGA-to-CCLE generalization. Positive x axis values indicate better performance for the best model, and negative values indicate better performance for the smallest good model.

(C) Distribution of performance comparisons between best and smallest good model selection strategies for CCLE-to-TCGA generalization.

(D) *NF1* mutation status prediction performance generalizing from TCGA (holdout, orange), to CCLE (green), with the best and smallest good models labeled. Shading represents 95% confidence intervals derived from 1,000 bootstrap samples at each tested level of regularization.

(E) *PIK3CA* mutation status prediction performance generalizing from TCGA (holdout, orange) to CCLE (green), with the best and smallest good models labeled.

lines for *IDH1* mutation classifiers.[24] For generalization from CCLE to TCGA, we observed a more pronounced upward shift toward better performance on CCLE and worse on TCGA, with most genes performing better on the CCLE holdout data and very few genes generalizing comparably to TCGA samples (Figure 2D).

"Best" and "smallest good" model selection strategies perform comparably

To address the question of whether sparser or more parsimonious models tend to generalize better or not, we implemented two model selection schemes and compared them for TCGA-to-CCLE and CCLE-to-TCGA mutation prediction problems (Figure 3A). The best model selection scheme chooses the top-performing model (LASSO parameter) on the holdout dataset from the same source as the training data and applies it to the test data from the other data source. The intention of the smallest good model selection scheme is to balance parsimony with reasonable performance on the holdout data since simply selecting the smallest possible model (generally, the dummy regressor/mean predictor) is not likely to generalize well.

To accomplish this, we rely on the "*lambda.1se*" heuristic used in the *glmnet* R package for generalized linear models, which is one of the default methods for parameter choice and model selection.[25] We first identify models with performance within one standard error of the top-performing model on the

holdout dataset. Then, from this subset of relatively well-performing models, we choose the smallest (i.e., strongest LASSO penalty) to apply to the test data. In both cases, we exclusively use the holdout data to select a model and only apply the model to out-of-dataset samples to evaluate generalization performance *after* model selection. Applying these criteria to both TCGA-to-CCLE and CCLE-to-TCGA prediction problems, we saw that model sizes (number of nonzero gene expression features) tended to differ by approximately an order of magnitude between model selection approaches, with medians on the order of 100 nonzero features for the best models and on the order of 10 nonzero features for the smallest good models (Figure S4). Still, there was considerable variation between target genes, and some best-performing models included substantially more features than the median, including classifiers we have previously observed to perform well such as *TP53*, *PTEN*, and *SETD2*.

For TCGA-to-CCLE generalization, 37/71 genes (52.1%) had better performance for the best model, and 24/71 genes (33.8%) had better generalization performance with the smallest good model. The other 10 genes had the same best and smallest good model performances: in other words, the smallest good model was also the best performing overall, so the performance difference between the two was exactly 0 (Figure 3B). For CCLE-to-TCGA generalization, 30/66 genes (45.5%) had better performance for the best model and 25/66 (37.9%) for the smallest good, with the other 11 having the same model fulfill both criteria (Figure 3C). Overall, these results do not support the hypothesis that the most parsimonious model generalizes the best: for both generalization problems, there are slightly more genes where the best-performing model on the holdout dataset is also the best-performing on the test set, although there are some genes where the smallest good approach works well (CCLE-to-TCGA Wilcoxon signed-rank $p = 0.721$, TCGA-to-CCLE Wilcoxon signed-rank $p = 0.963$).

We examined genes that fell into either category for TCGA-to-CCLE generalization (dotted lines on Figure 3B). For *NF1*, the best model outperforms the smallest good model (Figure 3D). Comparing holdout (orange) and cross-dataset (green) performance, both generally follow a similar trend, with the cross-dataset performance near its peak when the holdout performance peaks at a regularization parameter of $\alpha = 0.01$. *PIK3CA* is an example of the opposite, a gene where the smallest good model tends to outperform the best model (Figure 3E). In this case, better cross-dataset performance occurs at a higher level of regularization (further left on the x axis), $\alpha = 0.019$, than the peak for the holdout performance, $\alpha = 0.027$. This suggests that a *PIK3CA* mutation status classifier that is more parsimonious but has slightly worse performance does tend to generalize more effectively across datasets from TCGA to CCLE.

Generalization across cancer types yields similar results to generalization across datasets

To evaluate generalization across biological contexts within a dataset, we trained mutation prediction classifiers on all but one cancer type in TCGA, performed model selection on a holdout set stratified by cancer type, and held out the remaining cancer type as a test set. We performed the same best vs. smallest good analysis that was previously described across 291 gene/holdout cancer type combinations (Figure 4A). We observed 135/291 gene/cancer type combinations (46.4%) that had better generalization performance with the best model compared to 130/291 (44.7%) for the smallest good model. The other 26 gene/cancer type combinations had the same best and smallest good model and thus no difference in performance. This is consistent with our cross-dataset experiments, with slightly more instances where the best model on the stratified holdout data also generalizes the best but no pronounced distributional shift in either direction (Wilcoxon signed-rank $p = 0.599$).

We looked in more detail at two examples of gene/cancer type combinations, one on either side of the 0 point for cross-cancer type generalization. For prediction of *PIK3CA* mutation status in rectal adenocarcinoma (READ), we observed the best cross-cancer type performance for relatively low levels of regularization/high x axis values at $\alpha = 0.027$ (Figure 4B). For prediction of *NF1* mutation status in uterine corpus endometrial carcinoma (UCEC), on the other hand, we observed the best cross-cancer generalization for a high level of regularization ($\alpha = 0.0072$), and generalization capability for the best parameter on the stratified holdout set ($\alpha = 0.01$) was lower (Figure 4C). It is also interesting to note that in the previous experiments generalizing from TCGA to CCLE, we used *PIK3CA* as an example of a gene where the smallest good model performs best and *NF1* as an example where the best model was selected, and this tendency was reversed for these two cancer types. This highlights the importance of considering generalization to the cancer type or sample cohort of interest independently of general trends for a particular classifier whenever possible.

We aggregated results across genes for each cancer type, looking at performance in the held-out cancer type compared to performance on the stratified holdout set (Figure 4D). Cancer types that were particularly difficult to generalize to (better performance on stratified data than cancer type holdout or positive y axis values) include testicular cancer (TGCT) and soft tissue sarcoma (SARC), which are notable because they are not carcinomas like the majority of cancer types included in TCGA, potentially making generalization harder. We also aggregated results across cancer types for each gene, identifying a distinct set of genes where classifiers tend to generalize poorly no matter what cancer type is held out (Figure S5). Included in this set of genes with poor generalization performance are *HRAS*, *NRAS*, and *BRAF*, suggesting that a classifier that combines mutations in Ras pathway genes into a single "pathway mutation status" label (as described in Way et al.[26] or using more general computational approaches such as those in Haan et al.[27] and Bakhtiar et al.[28]) could be a better approach than separate classifiers for each gene.

In the cancer type aggregation plot (Figure 4D), thyroid carcinoma (THCA) stood out as a carcinoma that had poor performance when held out. In our experiments, the only genes in which THCA is included as a held-out cancer type are *BRAF* and *NRAS*; generalization performance for both genes is below cross-validation performance but slightly worse for *NRAS* than *BRAF* (Figure S6). Previous work suggests that the *BRAF* mutation tends to have a different functional signature in THCA than other cancer types, and withholding THCA from the training set improved classifier performance, which could at least in part explain the difficulty of generalizing to THCA we observe.[26]

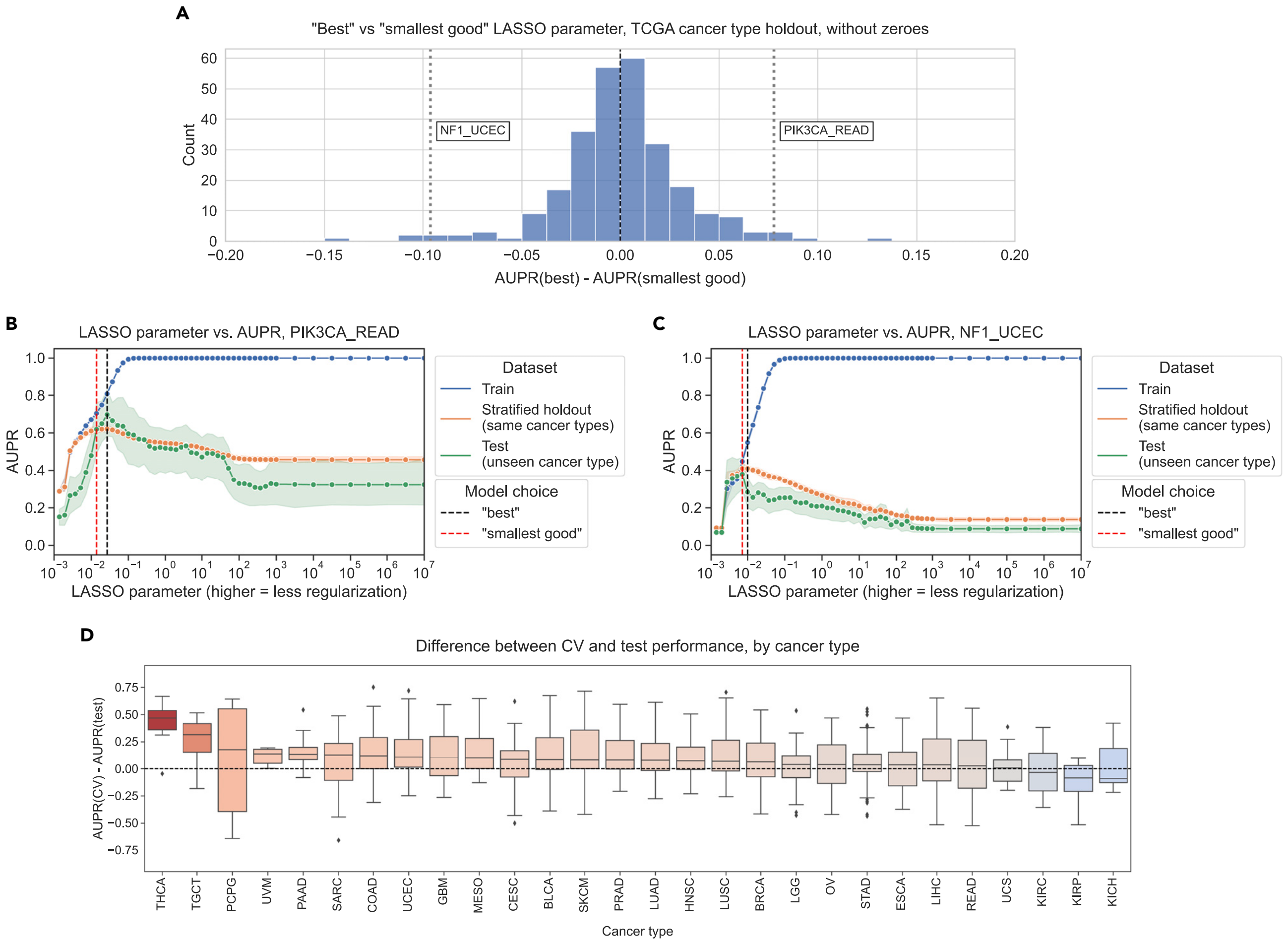

Figure 4. Evaluating generalization across cancer types

(A) Distribution of performance comparisons between best and smallest good model selection strategies for generalization across TCGA cancer types. Each point is a gene/cancer type combination; positive x axis values indicate better performance for the best model, and negative values indicate better performance for the smallest good model.

(B) *PIK3CA* mutation status prediction performance generalizing from other cancer types in TCGA (stratified holdout, orange) to rectal adenocarcinoma (READ; green), with the best and smallest good models labeled. Shading represents 95% confidence intervals derived from 1,000 bootstrap samples at each tested level of regularization.

(C) *NF1* mutation status prediction performance generalizing from other cancer types in TCGA (stratified holdout, orange) to uterine corpus endometrial carcinoma (UCEC; green), with best and smallest good models labeled.

(D) Distributions of performance difference between CV data (same cancer types as train data) and holdout data (cancer types not represented in train data), by held-out cancer type, after correcting for baseline mutation frequency in each cancer type. Each point is a gene whose mutation status classifier was used to make predictions on out-of-dataset samples in the relevant cancer type.

Restricting neural network hidden layer size does not improve generalization

To test whether or not findings generalize to non-linear models, we trained a 3-layer neural network to predict mutation status from gene expression for generalization from TCGA to CCLE, and we varied the size of the first hidden layer to control regularization/model complexity. We fixed the size of the second hidden layer to be half the size of the first layer, rounded up to the nearest integer; further details are provided in methods. For *EGFR* mutation status prediction, we saw that performance for small hidden layer sizes was noisy but generally lower than for higher hidden layer sizes on train, holdout, and test sets, reflecting "underfitting" or high bias (Figure 5A). On average, over all 71 genes from Vogelstein et al., performance on both held-out TCGA data and CCLE data tends to increase until a hidden layer size of 10–50, then flattens (Figure 5B). To explore additional approaches to neural network regularization, we also tried varying the dropout and weight decay for *EGFR* and *KRAS* mutation status classification while holding the hidden layer size constant. The results followed a similar trend, with generalization performance generally tracking performance on holdout data (Figure S7). We also preprocessed the input gene expression features using principal-component analysis (PCA) and varied the number of PCA features retained as input to the neural network; for *EGFR*, the best generalization performance and holdout performance both occurred at 1,000 PCs, but for *KRAS*, the model

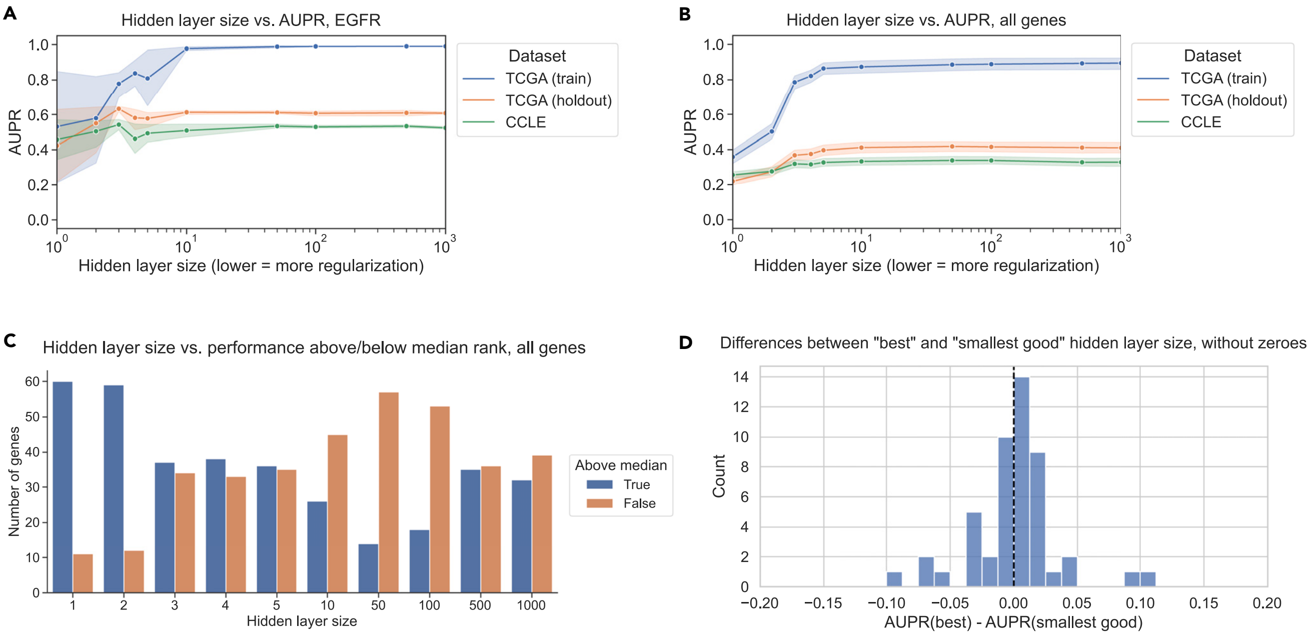

Figure 5. Model complexity and generalization in neural network models

(A) *EGFR* mutation status prediction performance on training samples from TCGA (blue), held-out TCGA samples (orange), and CCLE samples (green) across varying neural network hidden layer sizes. Shading represents 95% confidence intervals derived from 1,000 bootstrap samples at each tested hidden layer size.
(B) Mutation status prediction performance summarized across all genes from Vogelstein et al. on training samples from TCGA (blue), held-out TCGA samples (orange), and CCLE samples (green) across varying neural network hidden layer sizes.
(C) Distribution of ranked performance values above/below the median rank for each gene for each of the hidden layer sizes evaluated. Lower ranks indicate better performance and higher ranks indicate worse performance relative to other hidden layer sizes.
(D) Distribution of performance comparisons between best and smallest good model selection strategies for TCGA-to-CCLE generalization with neural network hidden layer size as the regularization axis. Positive x axis values indicate better performance for the best model, and negative values indicate better performance for the smallest good model.

generalized better to cell line data for fewer PCs than its peak holdout performance (Figure S8).

It can be challenging to measure which hidden layer sizes tended to perform relatively well or poorly across classifiers, as different genes may have different baseline performance AUPR values and overall classifier effect sizes. In order to summarize across genes, for each gene, we ranked the range of hidden layer sizes by the corresponding models' generalization performance on CCLE (Figure 5C). Concretely, for a particular hidden layer size, low ranks represent good performance, and high ranks represent poor performance. We then visualized the distribution of ranks above and below the median rank of 5.5/10 for each hidden layer size across all genes. In summary, for a given hidden layer size, a high proportion of ranks above the median (true, or blue bar, Figure 5C) signifies poor overall performance for that hidden layer size, and a high proportion of ranks below the median (false, or orange bar, Figure 5C) signifies good performance. We saw that small hidden layer sizes tended to generalize poorly (<5 but most pronounced for 1 and 2), and intermediate hidden layer sizes tended to generalize well (10–100 and sometimes 500/1,000). This suggests that some degree of parsimony or simplicity could be useful but that very simple models do not tend to generalize well.

We also performed the same best/smallest good analysis as with the linear models, using hidden layer size as the regularization axis instead of LASSO regularization strength. We observed a distribution centered around 0, suggesting that the best and smallest good models tend to generalize similarly (Figure 5D). 28/71 genes (45.2%) had better generalization performance with the best model compared to 21/71 (28.6%) for the smallest good model and 22 with the same best and smallest good model. We extended our analyses to two additional non-linear model classes as well, for TCGA-to-CCLE generalization: XGBoost gradient boosting classification and a deeper neural network with 5 hidden layers. For XGBoost, using the *n_estimators* (number of tree estimators to combine) and *max_depth* (maximum depth of each tree) parameters to control model complexity, we saw a similar relationship between holdout performance on TCGA and generalization performance on CCLE to that of the LASSO experiments, although model performance was generally more stable across parameter settings (Figure S9). For the 5-layer neural networks, the generalization results were similar to the 3-layer neural networks, although underfitting/high bias was more obvious for very small hidden layer sizes, and there was a slightly more pronounced preference for larger hidden layer sizes overall (Figure S10).

DISCUSSION

Using public cancer genomics and transcriptomics data from TCGA and CCLE, we studied the generalization of mutation status classifiers for a wide variety of cancer driver genes. We

designed experiments to evaluate generalization across biological contexts by holding out cancer types in TCGA and across datasets by training models on TCGA and evaluating them on CCLE, and vice versa. We found that, in general, smaller or more parsimonious models do not tend to generalize more effectively across cancer types or across datasets, and in the absence of prior knowledge about a prediction problem, simply choosing the model that performs the best on a holdout dataset is at least as effective for selecting models that generalize. Given that similar smallest good heuristics are used broadly across genomics studies (see, e.g., Wang et al.,[29] Shao et al.,[30] and Li et al.[31]), we expect these results to have implications on current practices.

Our results were similar in both linear models (LASSO logistic regression) and non-linear deep neural networks when using hidden layer size as the regularization parameter of interest. In our non-linear model experiments, we did not observe better generalization across datasets for fully connected neural networks with fewer hidden layer nodes, and our preliminary results indicated a similar trend for dropout and weight decay. Compared to linear models, it is less clear how to define a small or parsimonious neural network model since there are many regularization techniques that one may use to control complexity. Rather than simply removing nodes and keeping the network fully connected, another approach to parsimony could be to select an inductive bias to guide the size reduction of the network. Existing examples include network structures guided by protein-protein interaction networks or function/pathway ontologies.[32–35] It is possible that a smaller neural network with a structure that corresponds more appropriately to the prediction problem would achieve better generalization results, although choosing an apt network structure or data source can be a challenging aspect of such efforts.

For generalization from CCLE to TCGA, we observed that performance was generally worse on human tumor samples from TCGA than for held-out cell lines. This could, at least in part, be a function of sample size: the number of cell lines in CCLE is approximately an order of magnitude smaller than the number of tumor samples in TCGA (~10,000 samples in TCGA vs. ~1,500 cell lines in CCLE, although the exact number of samples used to train and evaluate our classifiers varies by gene; see methods for further details). There are also plausible biological and technical explanations for the difficulty of generalizing to human tumor samples. This result could reflect the imperfect and limited nature of cancer cell lines as a model system for human tumors, which previous studies have pointed out.[36–38] In addition, the CCLE data are collected and processed uniformly, as described in Ghandi et al.,[20] while TCGA data are processed by a uniform pipeline but collected from a wide variety of different cancer centers around the US.[19]

When we ranked cancer types in order of their generalization difficulty aggregated across genes, we noticed a slight tendency toward non-carcinoma cancer types (testicular cancer [TGCT], soft tissue sarcoma [SARC], skin cutaneous melanoma [SKCM]) being difficult to generalize to. It has been pointed out in other biological data types that holding out entire contexts or domains is necessary for a full picture of generalization performance,[39,40] which our results corroborate. This highlights a potential weakness of using TCGA's carcinoma-dominant pan-cancer data as a training set for a broad range of tasks, for instance in foundation models, which are becoming feasible for some genomics applications.[41–43] One caveat of our analysis is that each cancer type is included in the training data or held out for a different subset of genes, so it is difficult to detangle gene-specific effects (some mutations have less distinguishable functional effects on gene expression than others) from cancer-type-specific effects (some cancer types are less similar to each other than others) on prediction performance using our experimental design.

Other aspects of TCGA that may make it less representative for certain prediction problems is that it is composed of primary tumor samples from adult patients with relatively high quality (fresh frozen, generally high purity, although this varies by tissue[44]), so it is possible that generalization to metastatic samples, pediatric patients, or lower-quality (e.g., formalin-fixed paraffin-embedded [FFPE]) clinical samples would present different properties. Similarly, mutation calling in CCLE cell lines is limited by the lack of a matched normal reference, although we generally observed reasonable generalization to cell lines, suggesting that the quality of mutation calls is likely adequate in the genes we considered. Overall, however, we believe the size and tissue representations of TCGA and CCLE make them apt benchmarks for model performance in cancer -omics.

METHODS

Mutation data download and preprocessing

To generate binary mutated/non-mutated gene labels for our machine learning model, we used mutation calls for TCGA samples from MC3[45] and copy-number threshold calls from GISTIC2.0.[46] MC3 mutation calls were downloaded from the Genomic Data Commons (GDC) of the National Cancer Institute at https://gdc.cancer.gov/about-data/publications/pancanatlas. Thresholded copy-number calls are from an older version of the GDC data and are available here: https://figshare.com/articles/dataset/TCGA_PanCanAtlas_Copy_Number_Data/6144122. We removed hypermutated samples, defined as two or more standard deviations above the mean non-silent somatic mutation count, from our dataset to reduce the number of false positives (i.e., non-driver mutations). Any sample with either a non-silent somatic variant or a copy-number variation (CNV; copy-number gain in the target gene for oncogenes and copy-number loss in the target gene for tumor-suppressor genes) was included in the positive set; all remaining samples were considered negative for mutation in the target gene.

We followed a similar procedure to generate binary labels for cell lines from CCLE using the data available on the DepMap download portal at https://depmap.org/portal/download/all/. Mutation information was retrieved from the OmicsSomaticMutations.csv data file, and copy-number information was retrieved from the OmicsCNGene.csv data file, both from the 22Q2 public release. We thresholded the CNV log ratios provided by CCLE into binary gain/loss calls using a lower threshold of $\log_2(3/2)$ (i.e., cell lines with a log ratio below this threshold were considered to have a full copy loss in the corresponding gene) and an upper threshold of $\log_2(5/2)$ (i.e., cell lines with a log ratio above this threshold were considered to have a full copy gain in the corresponding gene). After applying the same hypermutation

criteria that we used for TCGA, no cell lines in CCLE were identified as hypermutated. After preprocessing, 1,402 cell lines with mutation and copy-number data remained. We then combined non-silent point mutations and copy-number gain/loss information into binary labels using the same criteria as for TCGA.

Gene expression data download and preprocessing

RNA-seq data for TCGA were downloaded from GDC at the same link provided above for the Pan-Cancer Atlas. We discarded non-protein-coding genes and genes that failed to map and removed tumors that were measured from multiple sites. After filtering to remove hypermutated samples and taking the intersection of samples with both mutation and gene expression data, 9,074 TCGA samples remained.

RNA-seq data for CCLE were downloaded from the DepMap download portal in the CCLE_expression.csv data file from the 22Q2 public release. After taking the intersection of CCLE cell lines with both mutation and gene expression data, 1,402 cell lines remained. For experiments making predictions across datasets (i.e., training models on TCGA and evaluating performance on CCLE, or vice versa), we took the intersection of genes in both datasets, resulting in 16,041 gene features. For experiments where only TCGA data were used (i.e., evaluating models on held-out cancer types), we used all 16,148 gene features present in TCGA after the filtering described above.

Cancer gene set construction

In order to study mutation status classification for a diverse set of cancer driver genes, we started with the set of 125 frequently altered genes from Vogelstein et al.[21] (all genes from Table S2A in that study). For each target gene, to ensure that the training dataset was reasonably balanced (i.e., that there would be enough mutated samples to train an effective classifier), we included only cancer types with at least 15 mutated samples and at least 5% mutated samples, which we refer to here as valid cancer types. In some cases, this resulted in genes with no valid cancer types, which we dropped from the analysis. Out of the 125 genes originally listed in the Vogelstein et al. cancer gene set, we retained 71 target genes for TCGA-to-CCLE analysis and 66 genes for CCLE-to-TCGA analyses. For these analyses, each gene needed at least one valid cancer type in TCGA and one valid cancer type in CCLE to construct the train and test sets. For the cancer type holdout analysis, we retained 56 target genes: in this case, each gene needed at least two valid cancer types in TCGA to be retained, one to train on and one to hold out.

Classifier setup and cross-validation design

We trained logistic regression classifiers to predict whether or not a given sample had a mutational event in a given target gene using gene expression features as explanatory variables. Our model was trained on gene expression data (X) to predict somatic mutation presence or absence (y) in a target gene. To control for varying mutation burden per sample, we included $\log_{10}$(sample mutation count) in our models as a covariate. Since gene expression datasets tend to have many dimensions and comparatively few samples, we used a LASSO penalty to perform feature selection.[47] LASSO logistic regression has the ability to generate sparse models (some or most coefficients are 0), as well as having a single tunable hyperparameter which can be easily interpreted as an indicator of regularization strength/model simplicity.

LASSO (L_1-penalized) logistic regression finds the feature weights $\widehat{w} \in \mathbb{R}^p$, solving the following optimization problem:

$$\widehat{w} = \text{argmin}_w \left(C \cdot l(X, y; w)\right) + \|w\|_1,$$

where $i \in \{1, \ldots, n\}$ denotes a sample in the dataset, $X_i \in \mathbb{R}^p$ denotes features (gene expression measurements) from the given sample, $y_i \in \{0, 1\}$ denotes the label (mutation presence/absence) for the given sample, and $l(\cdot)$ denotes the negative log likelihood of the observed data given a particular choice of feature weights, i.e.,

$$l(X, y; w) = -\sum_{i=1}^{n} y_i \log\left(\frac{1}{1+e^{-w^\top X_i}}\right) + (1 - y_i)\log\left(1 - \frac{1}{1+e^{-w^\top X_i}}\right).$$

Given weight values $\widehat{w}$, it is straightforward to predict the probability of a positive label (mutation in the target gene) $P(y^* = 1 | X^*; \widehat{w})$ for a test sample X^*:

$$P(y^* = 1 | X^*; \widehat{w}) = \frac{1}{1+e^{-\widehat{w}^\top X^*}},$$

and the probability of no mutation in the target gene, $P(y^* = 0 | X^*; \widehat{w})$, is given by (1 − the above quantity).

This optimization problem leaves one hyperparameter to select: C, which controls the inverse of the strength of the L_1 penalty on the weight values (i.e., regularization strength scales with $\frac{1}{C}$). Although the LASSO optimization problem does not have a closed form solution, the loss function is convex, and iterative optimization algorithms are commonly used for finding reasonable solutions. For fixed values of C, we solved for $\widehat{w}$ using *scikit-learn*'s *LogisticRegression* method,[48] which uses the coordinate descent optimization method implemented in *liblinear*.[49] We selected this implementation rather than the *SGDClassifier* stochastic gradient descent implementation because coordinate descent/*liblinear* tends to generate sparser models and does not depend on a learning rate parameter, although after hyperparameter tuning, performance is generally comparable between the implementations.[50]

To assess model selection across contexts (datasets and cancer types), we trained models using a variety of LASSO parameters on 75% of the training dataset, holding out 25% of the training dataset as the cross-validation set and also evaluating across contexts as the test set. We trained models using C values evenly spaced on a dense logarithmic scale between (10^{-3} and 10^3), which was where we generally observed that performance varied the most, and a sparser logarithmic scale between (10^3 and 10^7) in order to capture models with very little regularization that included all features. In other words, the exact range we used is the output of the command: *numpy.concatenate(numpy.logspace(−3, 3, 43), numpy.logspace(3, 7, 21))*.

This range of regularization strength/sparsity levels was intended to give evenly distributed coverage across genes and cancer types that included underfit models (predicting only the

mean or using very few features, poor performance on all datasets), overfit models (performing perfectly on training data but comparatively poorly on cross-validation and test data), and a wide variety of models in between that typically included the best fits to the cross-validation and test data. To assess variability between train/CV splits, we used all 4 splits (25% holdout sets) × 2 random seeds for a total of 8 different training sets for each gene, using the same test set (i.e., all of the held-out context, either one cancer type or one dataset) in each case.

Best model vs. smallest good model analysis

For the best vs. smallest good model selection comparison, we started with 8 performance measurements (4 cross-validation folds × 2 random seeds) for each LASSO parameter. We took the mean over these 8 measurements to get a single performance measurement for each model (LASSO parameter) on the holdout dataset, which has the same composition as the training set. We used these per-parameter mean performance measurements to select the best model (LASSO parameter with the best mean performance on the holdout dataset) and the smallest good model (strongest LASSO parameter with mean performance within 1 standard error of the best mean performance value on the holdout dataset, as implemented in the *glmnet* R package's *lambda.1se* model selection method[25]). For the distributions of differences shown in the results, we took the difference in mean performance for the best and smallest good models for each gene, with positive differences indicating better performance for the best model and negative differences better performance for the smallest good model. Note that in each case, we are comparing model selection procedures for models trained on the same data (same training set/cross-validation split) and measuring the difference in model performance between procedures, so correcting for the baseline AUPR is unnecessary here.

Neural network setup and parameter selection

As a trade-off between computational cost and ability to represent non-linear decision boundaries, inspired by the architecture of the intermediate-complexity model described in Heil et al.,[51] we trained a three-layer fully connected neural network with rectified linear unit (ReLU) non-linearities[52] to predict mutation status. For the experiments described in the results and discussions sections, we varied the size of the first hidden layer in the range {1, 2, 3, 4, 5, 10, 50, 100, 500, 1,000}. We fixed the size of the second hidden layer to be half of the size of the first hidden layer, rounded up to the nearest integer, and the size of the third hidden layer was the number of classes, 2 in our case. Our models were trained for 100 epochs of mini-batch stochastic gradient descent in PyTorch[53] using the Adam optimizer[54] and a fixed batch size of 50. To select the remaining hyperparameters for each hidden layer size, we performed a random search over 10 combinations, with a single train/test split stratified by cancer type, using the following hyperparameter ranges: learning rate {0.1, 0.01, 0.001, 5e−4, 1e−4}, dropout proportion {0.1, 0.5, 0.75}, and weight decay (L_2 penalty) {0, 0.1, 1, 10, 100}. We used the same train/cross-validation split strategy described above for one random seed and 4 cross-validation splits, generating 4 different performance measurements for each gene and hidden layer size.

Although L_1 regularization can be used to more directly induce model sparsity in convex settings, we note that using L_1 regularization to control model complexity in neural networks is considerably more complex. Simply adding an additional loss term is not enough to achieve convergence to a sparse solution; the problem requires special optimizers and is the subject of ongoing research (see, e.g., Yun et al.[55]). For this reason, we focused on controlling neural network model complexity via the size and number of hidden layers, as well as the other approaches described above.

For the *EGFR* gene, we also ran experiments where we varied the dropout proportion and the weight decay hyperparameter as the regularization axis and selected the remaining hyperparameters (including the hidden layer size) using a random search. In these cases, we used a fixed range for dropout of {0.0, 0.05, 0.125, 0.25, 0.375, 0.5, 0.625, 0.75, 0.875, 0.95} and a fixed range for weight decay of {0.0, 0.001, 0.005, 0.01, 0.05, 0.1, 0.2, 0.3, 0.4, 0.5, 0.75, 1.0, 10.0}. All neural network analyses were performed on a Ubuntu 18.04 machine with an NVIDIA RTX 2060 GPU.

RESOURCE AVAILABILITY

Lead contact

Requests for information on or further resources from this study should be directed to Casey S. Greene (casey.s.greene@cuanschutz.edu).

Materials availability

This study did not generate new materials or reagents.

Data and code availability

The data from TCGA analyzed during this study were previously published as part of TCGA Pan-Cancer Atlas project[19] and are available from the NIH NCI GDC. The data from CCLE analyzed during this study were previously published[20] and are available from the Broad Institute's DepMap Portal. Raw classification results, performance figures for all genes in the Vogelstein et al. dataset, and parameter selection results and performance comparisons for each individual gene in the best vs. smallest good analyses are available on Figshare under a CC0 license.[56]

Software developed and used in this manuscript is available on GitHub at https://github.com/greenelab/pancancer-evaluation/ and on Zenodo.[57] The scripts used to download and preprocess the datasets for this study are available at https://github.com/greenelab/pancancer-evaluation/tree/master/00_process_data. Scripts for TCGA-to-CCLE and CCLE-to-TCGA CCLE comparisons (Figures 2 and 3) and neural network experiments (Figure 5) are available in the https://github.com/greenelab/pancancer-evaluation/tree/master/08_cell_line_prediction directory. Scripts for TCGA cancer type comparisons (Figure 4) are available in the https://github.com/greenelab/pancancer-evaluation/tree/master/02_cancer_type_classification directory. All scripts are available under the open-source BSD 3-clause license.

This manuscript was written using Manubot[58] and is available on GitHub at https://github.com/greenelab/generalization-manuscript under the CC0-1.0 license.

ACKNOWLEDGMENTS

This research was supported in part by grants from the National Institutes of Health (R01 CA237170, R01 HD109765, and R01 HG010067). This research was supported in part by the University of Pittsburgh Center for Research Computing through the resources provided. Specifically, this work used the HTC cluster, which is supported by NIH award number S10OD028483.

AUTHOR CONTRIBUTIONS

Conceptualization, J.C. and C.S.G.; methodology, J.C., M.C., and C.S.G.; software, J.C.; visualization, J.C.; writing – original draft, J.C.; writing – review &

CellPress
OPEN ACCESS

editing, J.C., M.C., and C.S.G.; funding acquisition, C.S.G.; supervision, C.S.G. All authors read and approved the final manuscript.

DECLARATION OF INTERESTS

During the manuscript revision, J.C. was employed at Repare Therapeutics.

SUPPLEMENTAL INFORMATION

Supplemental information can be found online at https://doi.org/10.1016/j.patter.2024.101115.

Received: January 2, 2024
Revised: August 1, 2024
Accepted: November 13, 2024
Published: December 6, 2024

REFERENCES

1. Antoniadis, A., Lambert-Lacroix, S., and Leblanc, F. (2003). Effective dimension reduction methods for tumor classification using gene expression data. Bioinformatics *19*, 563–570. https://doi.org/10.1093/bioinformatics/btg062.
2. Townes, F.W., Hicks, S.C., Aryee, M.J., and Irizarry, R.A. (2019). Feature selection and dimension reduction for single-cell RNA-Seq based on a multinomial model. Genome Biol. *20*, 295. https://doi.org/10.1186/s13059-019-1861-6.
3. Cieślik, M., and Chinnaiyan, A.M. (2017). Cancer transcriptome profiling at the juncture of clinical translation. Nat. Rev. Genet. *19*, 93–109. https://doi.org/10.1038/nrg.2017.96.
4. Chibon, F. (2013). Cancer gene expression signatures – The rise and fall? Eur. J. Cancer *49*, 2000–2009. https://doi.org/10.1016/j.ejca.2013.02.021.
5. Chen, H.-Y., Yu, S.-L., Chen, C.-H., Chang, G.-C., Chen, C.-Y., Yuan, A., Cheng, C.-L., Wang, C.-H., Terng, H.-J., Kao, S.-F., et al. (2007). A Five-Gene Signature and Clinical Outcome in Non–Small-Cell Lung Cancer. N. Engl. J. Med. *356*, 11–20. https://doi.org/10.1056/nejmoa060096.
6. Landemaine, T., Jackson, A., Bellahcène, A., Rucci, N., Sin, S., Abad, B.M., Sierra, A., Boudinet, A., Guinebretière, J.M., Ricevuto, E., et al. (2008). A Six-Gene Signature Predicting Breast Cancer Lung Metastasis. Cancer Res. *68*, 6092–6099. https://doi.org/10.1158/0008-5472.can-08-0436.
7. Cardoso, F., van't Veer, L.J., Bogaerts, J., Slaets, L., Viale, G., Delaloge, S., Pierga, J.-Y., Brain, E., Causeret, S., DeLorenzi, M., et al. (2016). 70-Gene Signature as an Aid to Treatment Decisions in Early-Stage Breast Cancer. N. Engl. J. Med. *375*, 717–729. https://doi.org/10.1056/nejmoa1602253.
8. Slodkowska, E.A., and Ross, J.S. (2009). MammaPrint™ 70-gene signature: another milestone in personalized medical care for breast cancer patients. Expert Rev. Mol. Diagn. *9*, 417–422. https://doi.org/10.1586/erm.09.32.
9. Massagué, J. (2007). Sorting Out Breast-Cancer Gene Signatures. N. Engl. J. Med. *356*, 294–297. https://doi.org/10.1056/nejme068292.
10. Weigelt, B., Pusztai, L., Ashworth, A., and Reis-Filho, J.S. (2011). Challenges translating breast cancer gene signatures into the clinic. Nat. Rev. Clin. Oncol. *9*, 58–64. https://doi.org/10.1038/nrclinonc.2011.125.
11. Friedman, J., Hastie, T., and Tibshirani, R. (2010). Regularization Paths for Generalized Linear Models via Coordinate Descent. J. Stat. Softw. 33, 1–22.
12. Krstajic, D., Buturovic, L.J., Leahy, D.E., and Thomas, S. (2014). Cross-validation pitfalls when selecting and assessing regression and classification models. J. Cheminform. *6*, 10. https://doi.org/10.1186/1758-2946-6-10.
13. Altman, D.G., and Royston, P. (2000). What do we mean by validating a prognostic model? Stat. Med. *19*, 453–473. https://doi.org/10.1002/(sici)1097-0258(20000229)19:4<453::aid-sim350>3.0.co;2-5.
14. Boulesteix, A.-L., Strobl, C., Augustin, T., and Daumer, M. (2008). Evaluating Microarray-based Classifiers: An Overview. Cancer Inform. *6*, 77–97. https://doi.org/10.4137/cin.s408.
15. Kass, R.E., Caffo, B.S., Davidian, M., Meng, X.-L., Yu, B., and Reid, N. (2016). Ten Simple Rules for Effective Statistical Practice. PLoS Comput. Biol. *12*, e1004961. https://doi.org/10.1371/journal.pcbi.1004961.
16. Ma, J., Fong, S.H., Luo, Y., Bakkenist, C.J., Shen, J.P., Mourragui, S., Wessels, L.F.A., Hafner, M., Sharan, R., Peng, J., and Ideker, T. (2021). Few-shot learning creates predictive models of drug response that translate from high-throughput screens to individual patients. Nat. Cancer *2*, 233–244. https://doi.org/10.1038/s43018-020-00169-2.
17. Sharifi-Noghabi, H., Harjandi, P.A., Zolotareva, O., Collins, C.C., and Ester, M. (2021). Out-of-distribution generalization from labelled and unlabelled gene expression data for drug response prediction. Nat. Mach. Intell. *3*, 962–972. https://doi.org/10.1038/s42256-021-00408-w.
18. Mourragui, S.M.C., Loog, M., Vis, D.J., Moore, K., Manjon, A.G., van de Wiel, M.A., Reinders, M.J.T., and Wessels, L.F.A. (2021). Predicting patient response with models trained on cell lines and patient-derived xenografts by nonlinear transfer learning. Proc. Natl. Acad. Sci. USA *118*, e2106682118. https://doi.org/10.1073/pnas.2106682118.
19. Cancer Genome Atlas Research Network, Weinstein, J.N., Collisson, E.A., Mills, G.B., Shaw, K.R.M., Ozenberger, B.A., Ellrott, K., Shmulevich, I., Sander, C., and Stuart, J.M. (2013). The Cancer Genome Atlas Pan-Cancer analysis project. Nat. Genet. *45*, 1113–1120. https://doi.org/10.1038/ng.2764.
20. Ghandi, M., Huang, F.W., Jané-Valbuena, J., Kryukov, G.V., Lo, C.C., McDonald, E.R., III, Barretina, J., Gelfand, E.T., Bielski, C.M., Li, H., et al. (2019). Next-generation characterization of the Cancer Cell Line Encyclopedia. Nature *569*, 503–508. https://doi.org/10.1038/s41586-019-1186-3.
21. Vogelstein, B., Papadopoulos, N., Velculescu, V.E., Zhou, S., Diaz, L.A., Jr., and Kinzler, K.W. (2013). Cancer Genome Landscapes. Science *339*, 1546–1558. https://doi.org/10.1126/science.1235122.
22. da Cunha Santos, G., Shepherd, F.A., and Tsao, M.S. (2011). EGFR Mutations and Lung Cancer. Annu. Rev. Pathol. *6*, 49–69. https://doi.org/10.1146/annurev-pathol-011110-130206.
23. Liu, H., Zhang, B., and Sun, Z. (2020). Spectrum of EGFR aberrations and potential clinical implications: insights from integrative pan-cancer analysis. Cancer Commun. *40*, 43–59. https://doi.org/10.1002/cac2.12005.
24. Jones, L.E., Hilz, S., Grimmer, M.R., Mazor, T., Najac, C., Mukherjee, J., McKinney, A., Chow, T., Pieper, R.O., Ronen, S.M., et al. (2020). Patient-derived cells from recurrent tumors that model the evolution of IDH-mutant glioma. Neurooncol. Adv. *2*, vdaa088. https://doi.org/10.1093/noajnl/vdaa088.
25. Friedman, J., Hastie, T., and Tibshirani, R. (2010). Regularization Paths for Generalized Linear Models via Coordinate Descent. J. Stat. Soft. *33*, 1–22. https://doi.org/10.18637/jss.v033.i01.
26. Way, G.P., Sanchez-Vega, F., La, K., Armenia, J., Chatila, W.K., Luna, A., Sander, C., Cherniack, A.D., Mina, M., Ciriello, G., et al. (2018). Machine Learning Detects Pan-cancer Ras Pathway Activation in The Cancer Genome Atlas. Cell Rep. *23*, 172–180.e3. https://doi.org/10.1016/j.celrep.2018.03.046.
27. Haan, D., Tao, R., Friedl, V., Anastopoulos, I.N., Wong, C.K., Weinstein, A.S., and Stuart, J.M. (2019). Using Transcriptional Signatures to Find Cancer Drivers with LURE. In Pacific Symposium on Biocomputing 2020 (WORLD SCIENTIFIC), pp. 343–354. https://doi.org/10.1142/9789811215636_0031.
28. Bakhtiar, H., Helzer, K.T., Park, Y., Chen, Y., Rydzewski, N.R., Bootsma, M.L., Shi, Y., Harari, P.M., Sharifi, M., Sjöström, M., et al. (2022). Identification of phenocopies improves prediction of targeted therapy response over DNA mutations alone. npj Genom. Med. *7*, 58. https://doi.org/10.1038/s41525-022-00328-7.
29. Wang, L., Yang, Y., Feng, L., Tan, C., Ma, H., He, S., Lian, M., Wang, R., and Fang, J. (2021). A novel seven-gene panel predicts the sensitivity

and prognosis of head and neck squamous cell carcinoma treated with platinum-based radio(chemo)therapy. Eur. Arch. Oto-Rhino-Laryngol. *278*, 3523–3531. https://doi.org/10.1007/s00405-021-06717-5.

30. Shao, F., Wang, Z., and Wang, S. (2021). Identification of *MYCN*-Related Gene as a Potential Biomarker for Neuroblastoma Prognostic Model by Integrated Analysis and Quantitative Real-Time PCR. DNA Cell Biol. *40*, 332–347. https://doi.org/10.1089/dna.2020.6193.
31. Li, X., Zhang, H., Liu, J., Li, P., and Sun, Y. (2021). Five crucial prognostic-related autophagy genes stratified female breast cancer patients aged 40–60 years. BMC Bioinf. *22*, 580. https://doi.org/10.1186/s12859-021-04503-y.
32. Kuenzi, B.M., Park, J., Fong, S.H., Sanchez, K.S., Lee, J., Kreisberg, J.F., Ma, J., and Ideker, T. (2020). Predicting Drug Response and Synergy Using a Deep Learning Model of Human Cancer Cells. Cancer Cell *38*, 672–684.e6. https://doi.org/10.1016/j.ccell.2020.09.014.
33. Kulmanov, M., Khan, M.A., Hoehndorf, R., and Wren, J. (2018). DeepGO: predicting protein functions from sequence and interactions using a deep ontology-aware classifier. Bioinformatics *34*, 660–668. https://doi.org/10.1093/bioinformatics/btx624.
34. Fortelny, N., and Bock, C. (2020). Knowledge-primed neural networks enable biologically interpretable deep learning on single-cell sequencing data. Genome Biol. *21*, 190. https://doi.org/10.1186/s13059-020-02100-5.
35. Hao, Y., Romano, J.D., and Moore, J.H. (2022). Knowledge-guided deep learning models of drug toxicity improve interpretation. Patterns *3*, 100565. https://doi.org/10.1016/j.patter.2022.100565.
36. Gillet, J.-P., Varma, S., and Gottesman, M.M. (2013). The Clinical Relevance of Cancer Cell Lines. J. Natl. Cancer Inst. *105*, 452–458. https://doi.org/10.1093/jnci/djt007.
37. Wilding, J.L., and Bodmer, W.F. (2014). Cancer Cell Lines for Drug Discovery and Development. Cancer Res. *74*, 2377–2384. https://doi.org/10.1158/0008-5472.can-13-2971.
38. Iorio, F., Knijnenburg, T.A., Vis, D.J., Bignell, G.R., Menden, M.P., Schubert, M., Aben, N., Gonçalves, E., Barthorpe, S., Lightfoot, H., et al. (2016). A Landscape of Pharmacogenomic Interactions in Cancer. Cell *166*, 740–754. https://doi.org/10.1016/j.cell.2016.06.017.
39. Schreiber, J., Singh, R., Bilmes, J., and Noble, W.S. (2020). A pitfall for machine learning methods aiming to predict across cell types. Genome Biol. *21*, 282. https://doi.org/10.1186/s13059-020-02177-y.
40. Whalen, S., Schreiber, J., Noble, W.S., and Pollard, K.S. (2022). Navigating the pitfalls of applying machine learning in genomics. Nat. Rev. Genet. *23*, 169–181. https://doi.org/10.1038/s41576-021-00434-9.
41. Nguyen, E., Poli, M., Faizi, M., Thomas, A., Birch-Sykes, C., Wornow, M., Patel, A., Rabideau, C., Massaroli, S., Bengio, Y., et al. (2023). HyenaDNA: Long-Range Genomic Sequence Modeling at Single Nucleotide Resolution. Preprint at arXiv. https://doi.org/10.48550/arXiv.2306.15794.
42. Cui, H., Wang, C., Maan, H., Pang, K., Luo, F., and Wang, B. (2024). scGPT: Towards Building a Foundation Model for Single-Cell Multi-omics Using Generative AI. Nature Methods. https://doi.org/10.1038/s41592-024-02201-0.
43. Hao, M., Gong, J., Zeng, X., Liu, C., Guo, Y., Cheng, X., Wang, T., Ma, J., Song, L., and Zhang, X. (2024). Large Scale Foundation Model on Single-cell Transcriptomics. Nature Methods. https://doi.org/10.1038/s41592-024-02305-7.
44. Aran, D., Sirota, M., and Butte, A.J. (2015). Systematic pan-cancer analysis of tumour purity. Nat. Commun. *6*, 8971. https://doi.org/10.1038/ncomms9971.
45. Ellrott, K., Bailey, M.H., Saksena, G., Covington, K.R., Kandoth, C., Stewart, C., Hess, J., Ma, S., Chiotti, K.E., McLellan, M., et al. (2018). Scalable Open Science Approach for Mutation Calling of Tumor Exomes Using Multiple Genomic Pipelines. Cell Syst. *6*, 271–281.e7. https://doi.org/10.1016/j.cels.2018.03.002.
46. Mermel, C.H., Schumacher, S.E., Hill, B., Meyerson, M.L., Beroukhim, R., and Getz, G. (2011). GISTIC2.0 facilitates sensitive and confident localization of the targets of focal somatic copy-number alteration in human cancers. Genome Biol. *12*, R41. https://doi.org/10.1186/gb-2011-12-4-r41.
47. Tibshirani, R. (1996). Regression Shrinkage and Selection Via the Lasso. J. Roy. Stat. Soc. B Stat. Methodol. *58*, 267–288. https://doi.org/10.1111/j.2517-6161.1996.tb02080.x.
48. Pedregosa, F., Varoquaux, G., Gramfort, A., Michel, V., Thirion, B., Grisel, O., Blondel, M., Prettenhofer, P., Weiss, R., Dubourg, V., et al. (2011). Scikit-learn: Machine Learning in Python. J. Mach. Learn. Res. *12*, 2825–2830.
49. Fan, R.-E., Chang, K.-W., Hsieh, C.-J., Wang, X.-R., and Lin, C.-J. (2008). LIBLINEAR: A Library for Large Linear Classification. J. Mach. Learn. Res. *9*, 1871–1874.
50. Crawford, J., Chikina, M., and Greene, C.S. (2024). Optimizer's dilemma: optimization strongly influences model selection in transcriptomic prediction. Bioinformatics Advances. https://doi.org/10.1093/bioadv/vbae004.
51. Heil, B.J., Crawford, J., and Greene, C.S. (2023). The effect of non-linear signal in classification problems using gene expression. PLoS Comput. Biol. *19*, e1010984. https://doi.org/10.1371/journal.pcbi.1010984.
52. Nair, V., and Hinton, G.E. (2010). Rectified linear units improve restricted boltzmann machines. In Proceedings of the 27th International Conference on Machine Learning ICML'10 (Omnipress), pp. 807–814.
53. Paszke, A., Gross, S., Massa, F., Lerer, A., Bradbury, J., Chanan, G., Killeen, T., Lin, Z., Gimelshein, N., Antiga, L., et al. (2019). PyTorch: An Imperative Style, High-Performance Deep Learning Library. Preprint at arXiv. https://doi.org/10.48550/arXiv.1912.01703.
54. Kingma, D.P., and Ba, J. (2017). Adam: A Method for Stochastic Optimization. Preprint at arXiv. https://doi.org/10.48550/arXiv.1412.6980.
55. Yun, J., Lozano, A.C., and Yang, E. (2021). Adaptive proximal gradient methods for structured neural networks. In Proceedings of the 35th International Conference on Neural Information Processing Systems 1865, pp. 24365–24378.
56. Crawford, J. (2023). Supplementary Files for Generalization Manuscript (figshare). https://doi.org/10.6084/m9.figshare.23826450.
57. Crawford, J. (2024). greenelab/pancancer-evaluation: Publication release. Zenodo. https://doi.org/10.5281/zenodo.13960412.
58. Himmelstein, D.S., Rubinetti, V., Slochower, D.R., Hu, D., Malladi, V.S., Greene, C.S., and Gitter, A. (2019). Open collaborative writing with Manubot. PLoS Comput. Biol. *15*, e1007128. https://doi.org/10.1371/journal.pcbi.1007128.

Patterns, Volume *5*

Supplemental information

Best holdout assessment is sufficient

for cancer transcriptomic model selection

Jake Crawford, Maria Chikina, and Casey S. Greene

Supplementary Material

Supplementary Note S1

We were interested in exploring the extent to which excluding the target gene's expression profile from the input features affects performance, if at all. Additionally, since our labels include both point mutations and copy number changes, we sought to determine whether the answer to this question depends on the inclusion of copy number changes in the label set for a particular gene. To test this across driver genes, we calculated the contribution of single nucleotide variant (SNV) and copy number variant (CNV) changes to each gene's positively labeled sample set, and picked ten genes where CNV changes make up a relatively large proportion of positive labels, and ten genes where CNV changes make up a small proportion of positive labels. Genes where positive labels commonly result from CNV changes are as follows:

Gene	SNV sample count	SNV + CNV count	SNV / (SNV + CNV) ratio
BAP1	105	146	0.719
CDKN2A	288	1308	0.220
EGFR	192	444	0.432
ERBB2	129	440	0.293
GNAS	99	266	0.372
KDM6A	163	295	0.553
PDGFRA	131	235	0.557
PTEN	584	985	0.593
RB1	259	522	0.496
SMAD4	131	289	0.453

And genes where samples are rarely positively labeled based on CNV changes:

Gene	SNV sample count	SNV + CNV count	SNV / (SNV + CNV) ratio
ARID1A	588	629	0.934
ATRX	455	508	0.896
BRAF	569	605	0.940
CTNNB1	297	304	0.977
EP300	256	265	0.966
IDH1	414	415	0.997
NRAS	169	170	0.994
RNF43	152	157	0.968
SETD2	252	279	0.903
TP53	3305	3372	0.980

We also considered baseline model performance in the choice of these gene sets. If a gene has a very low or very high SNV / (SNV + CNV) ratio but the associated classifier generally performs poorly, we wouldn't expect to observe a performance change, regardless of the input features. For this experiment, the 20 genes we selected all had a reasonably high performance baseline, to maximize our ability to observe changes if they occur.

We visualized the mean difference in performance for the best-performing and "smallest good" models (LASSO parameters) with the "control" set of features, as compared to the best-performing and "smallest good" models with the "drop target" set of features (all of the gene expression features except the target gene), shown in Figure S1. In general, we do observe that performance tends to be better for the "control" models, although there are some exceptions (EGFR, ERBB2, PDGFRA, PTEN, EP300) where the "drop target" model actually performs slightly better. We do observe that there are some genes (BAP1, CDKN2A, KDM6A, RB1, ARID1A, ATRX) where performance decreases considerably when the target gene is not present in the feature set. For both the "best" and "smallest good" model selection approaches, this effect is slightly more consistent in the "frequent CNV" gene set than in the "rare CNV" gene set (mean control - drop target difference of 0.021/0.019 in the "frequent CNV" genes as compared to 0.009/0.004 in the "rare CNV" genes), but in both cases there is considerable variance between genes.

Based on these results, given the observation that the mean difference in model performance is fairly small in both "frequent CNV" and "rare CNV" cases, and for both model selection approaches, we conclude that combining point mutation and CNV data and including the target gene in the feature set are reasonable general rules for our pan-cancer and pan-gene study. In general, our focus is less on individual prediction performance and more on model complexity, which is another degree removed from the individual prediction performance. In addition, including the target gene would seem most likely to increase the benefit of smaller models, as the single-gene could be considered particularly information rich. While these results don't seem to heavily influence our experiment examining generality, the exceptions we noted above emphasize the importance of considering the biological context in applications to specific driver genes or prediction problems.

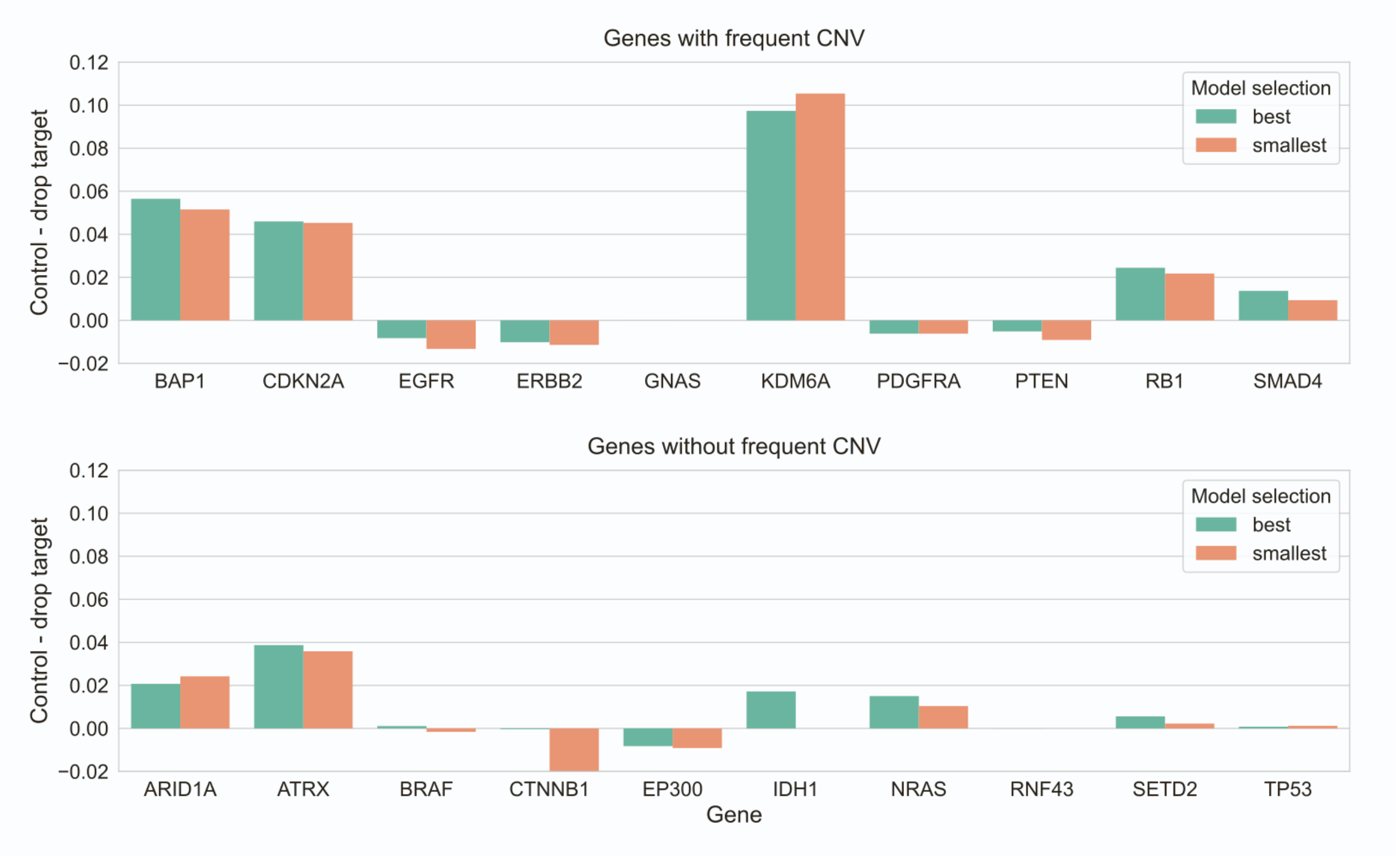

Figure S1: Bar plot showing difference in performance (AUPR) between models including and excluding the target gene, for genes where CNV changes are (top) and are not (bottom) frequently included in the label set, colored by model selection approach. Positive values represent better performance for the "control" model, and negative values better performance for the "drop target" model.

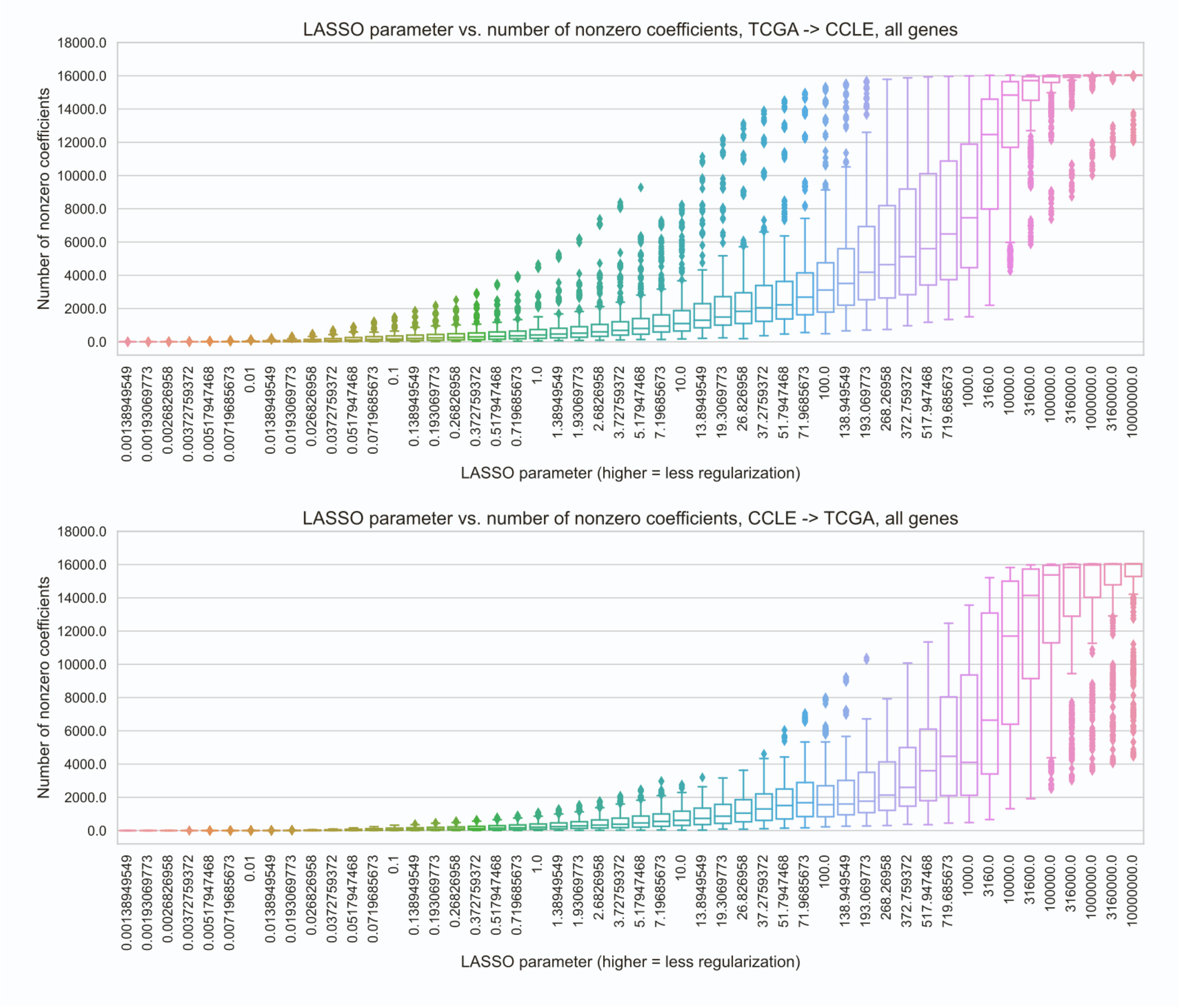

Figure S2: Number of nonzero coefficients (model sparsity) across varying regularization parameters, for 71 genes (TCGA to CCLE prediction, top) and 70 genes (CCLE to TCGA prediction, bottom) in the Vogelstein et al. dataset.

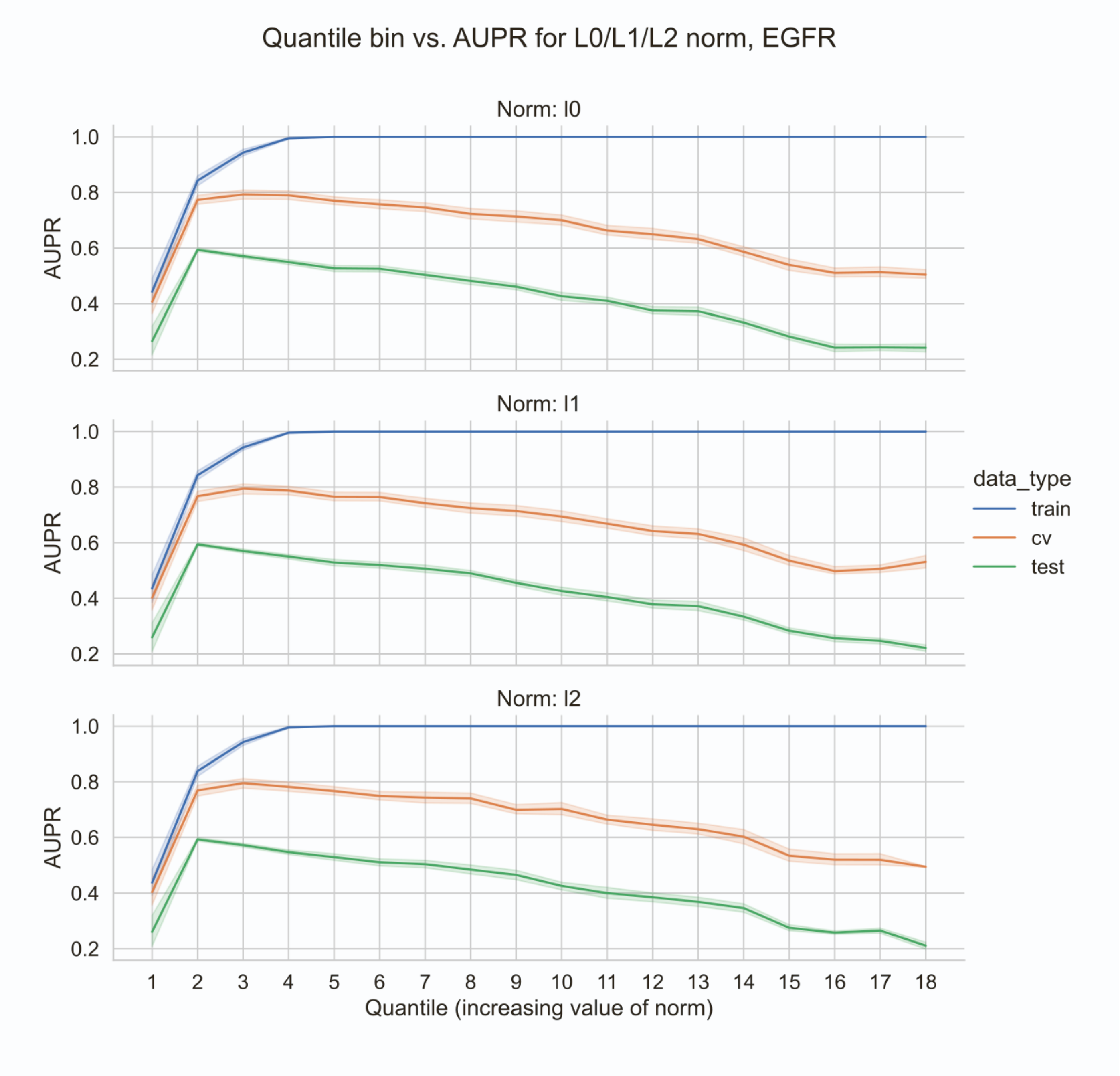

Figure S3: Value of norm of coefficient vector vs. performance, for EGFR mutation status prediction from TCGA to CCLE. The x-axis shows the value of each norm for each model, binned into quantiles in order to plot results on the same axis since each norm has a different scale.

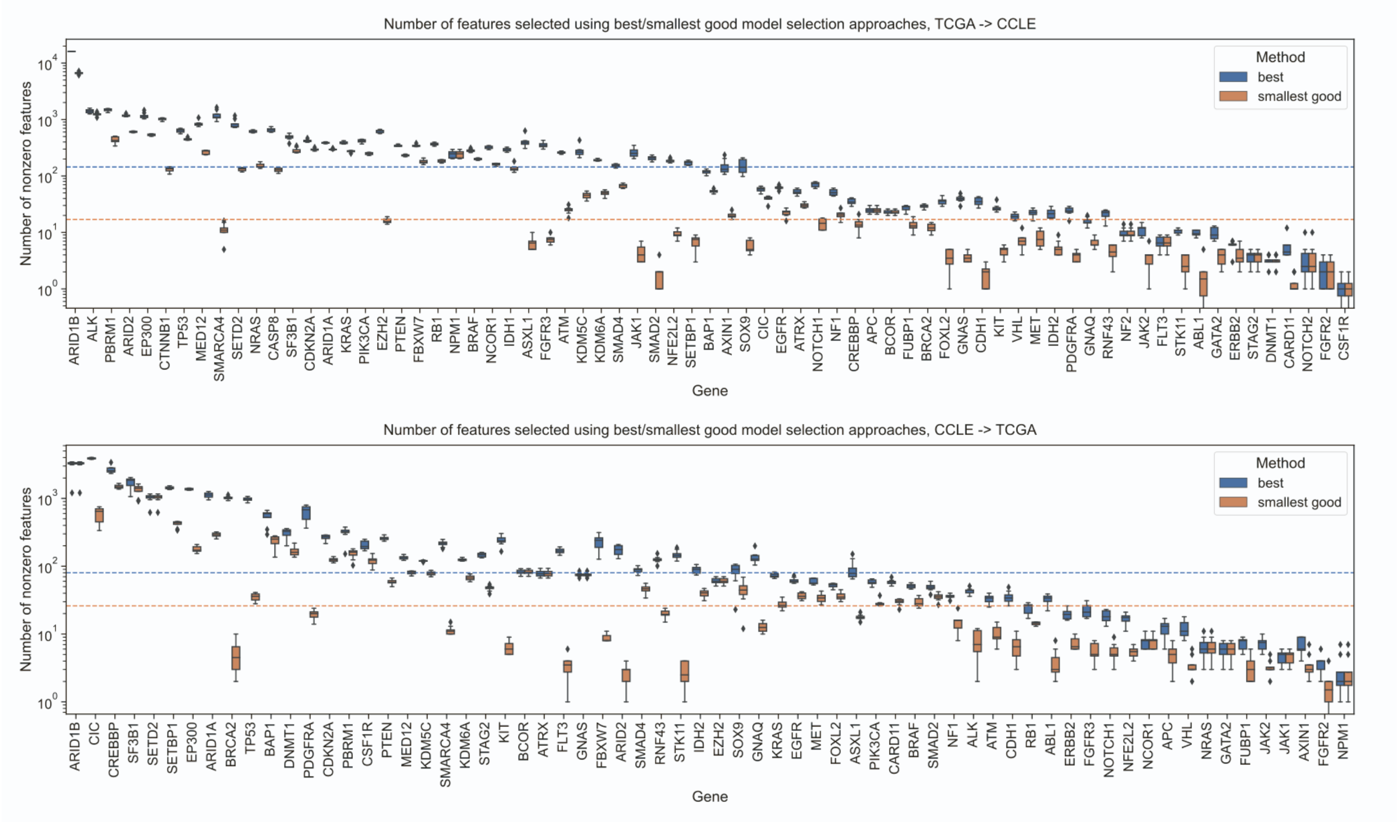

Figure S4: Distributions of number of features selected by the “best” and “smallest good” models, across seeds and folds, for TCGA to CCLE (top) and CCLE to TCGA (bottom) mutation prediction. Dotted lines show the median number of features for the best (blue) and smallest good (orange) numbers across genes: TCGA to CCLE - median of 144 features for the “best” approach and 17 features for the “smallest good” approach; CCLE to TCGA - median of 80 features for the “best” approach and 26 features for the “smallest good” approach.

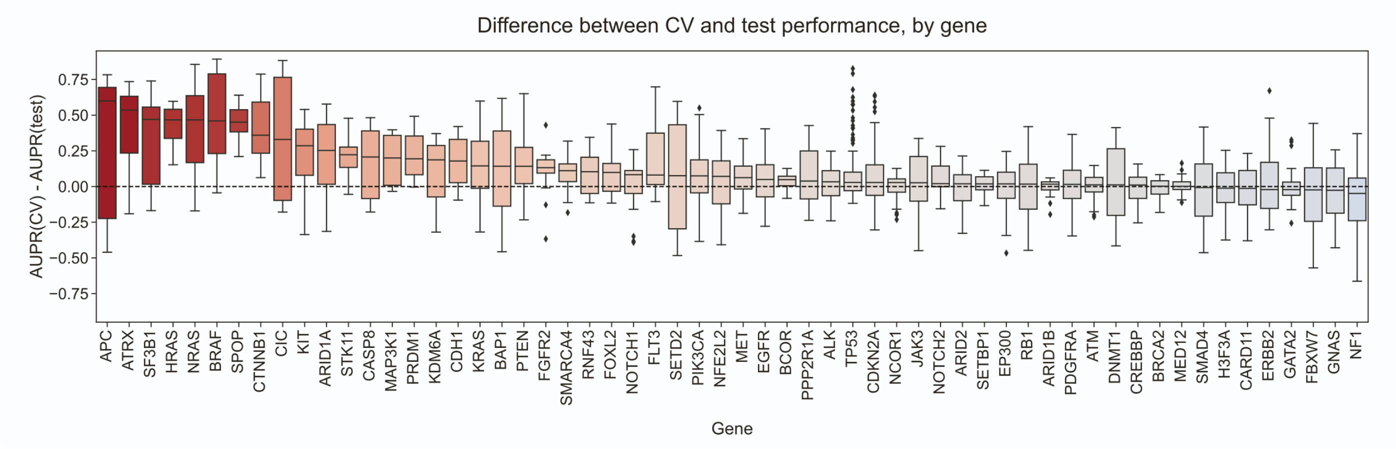

Figure S5: Distributions of performance difference between cross-validation data (same cancer types as training data) and holdout data (cancer types not represented in data), grouped by held-out gene. Each point shows performance for a single train/validation split for one cancer type that was held out, using a classifier trained to predict mutations in the given gene.

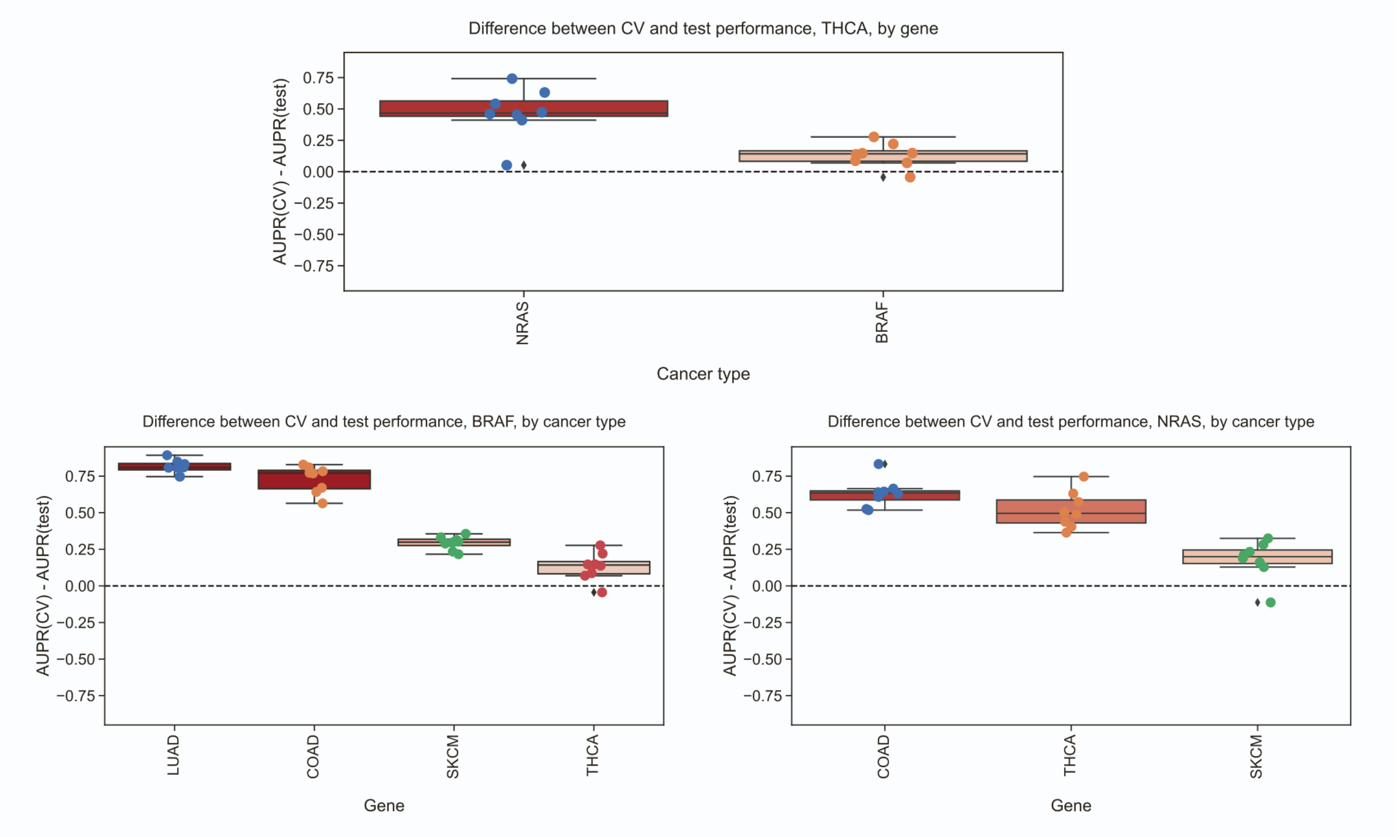

Figure S6: Top row: Distribution of performance differences when thyroid cancer (THCA) data is held out from training set across seeds/folds, grouped by gene. Bottom row: Distributions of performance differences for genes where THCA is included in training/holdout sets, relative to other cancer types that are included.

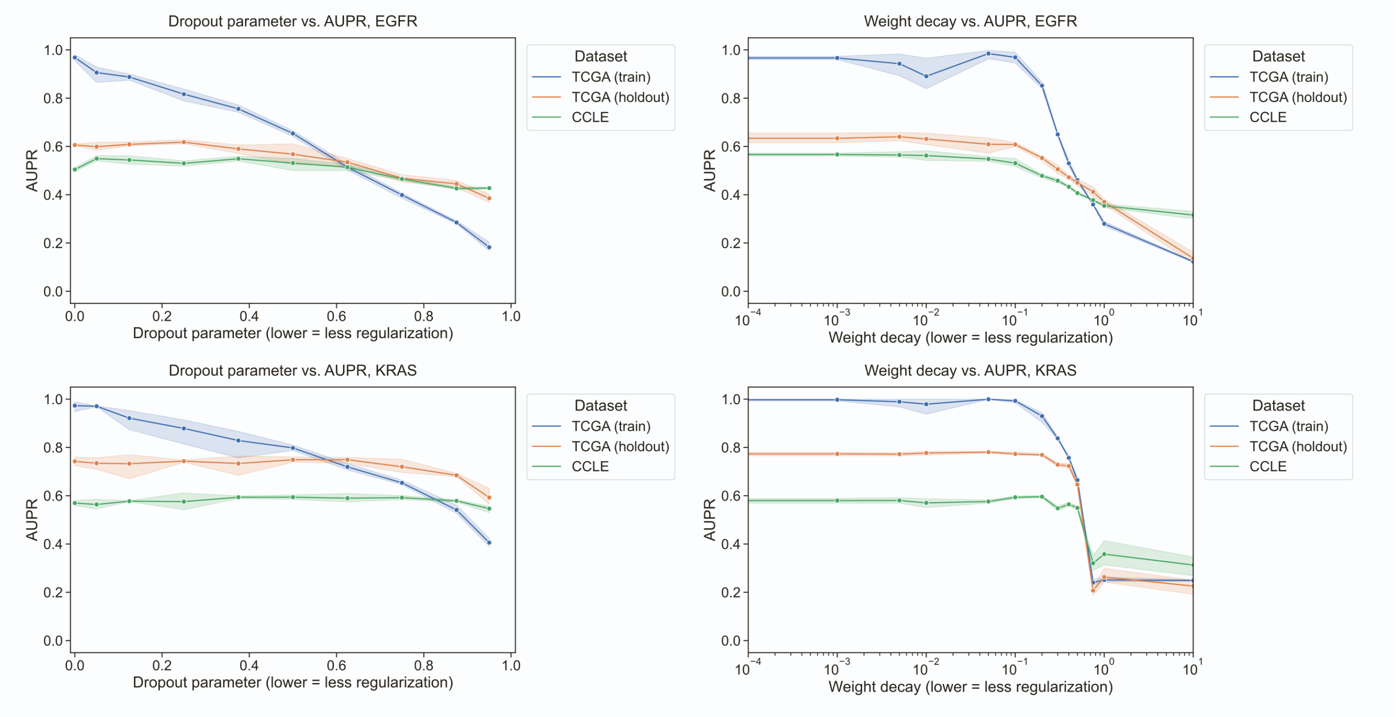

Figure S7: Performance vs. dropout parameter (first column) and weight decay strength (second column), for EGFR mutation prediction (first row) and KRAS mutation prediction (second row) using a 3-layer fully connected neural network trained on TCGA (blue/orange) and evaluated on CCLE (green).

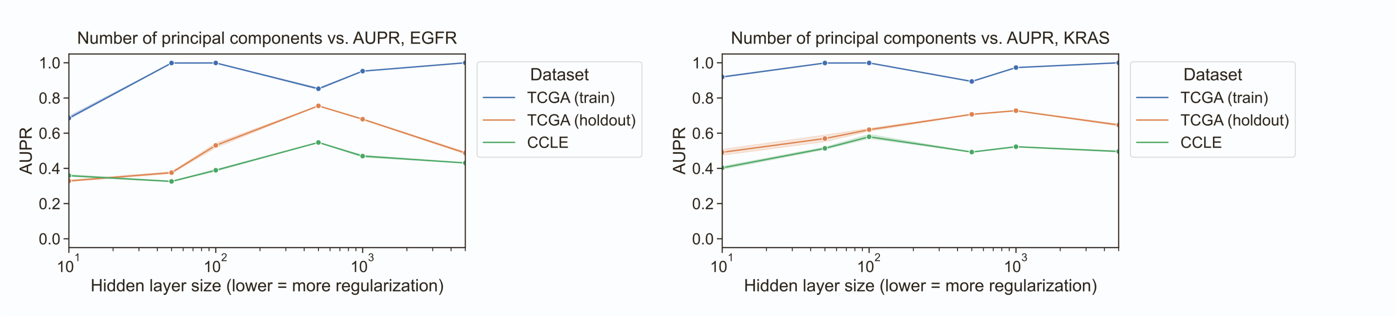

Figure S8: Performance vs. number of gene expression principal components, used as input to a 3-layer fully connected neural network trained on TCGA (blue/orange) and evaluated on CCLE (green), for EGFR and KRAS mutation status prediction.

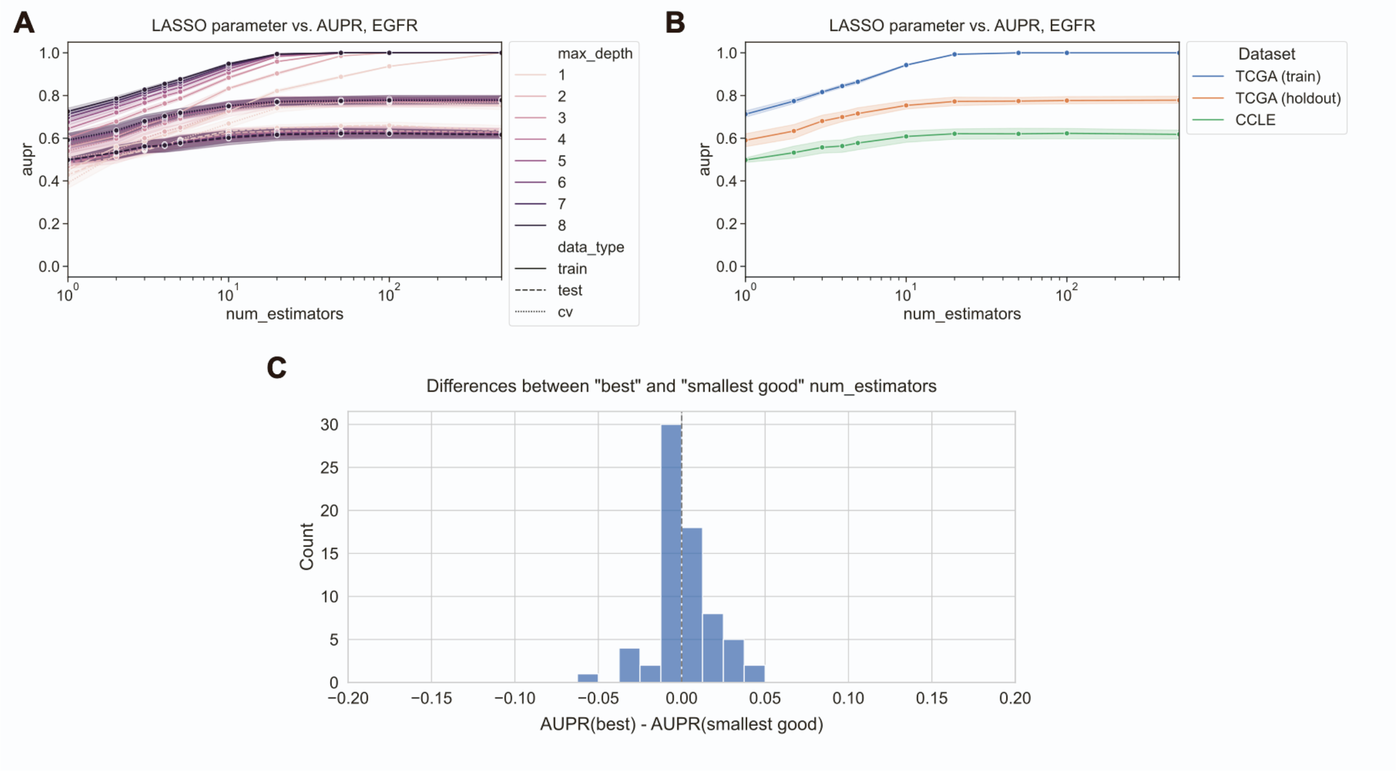

Figure S9: Performance across regularization parameter values for XGBoost mutation status classification, for generalization from TCGA to CCLE. Top row shows performance for EGFR across varying values of `num_estimators` and `max_depth` (Panel A), and for `max_depth=8` across a range of `num_estimators` (Panel B). Panel C summarizes the distribution of performance comparisons between "best" vs. "smallest good" `num_estimators` (33/71 genes best > smallest good, 17/71 smallest good > best, 20/71 best = smallest good).

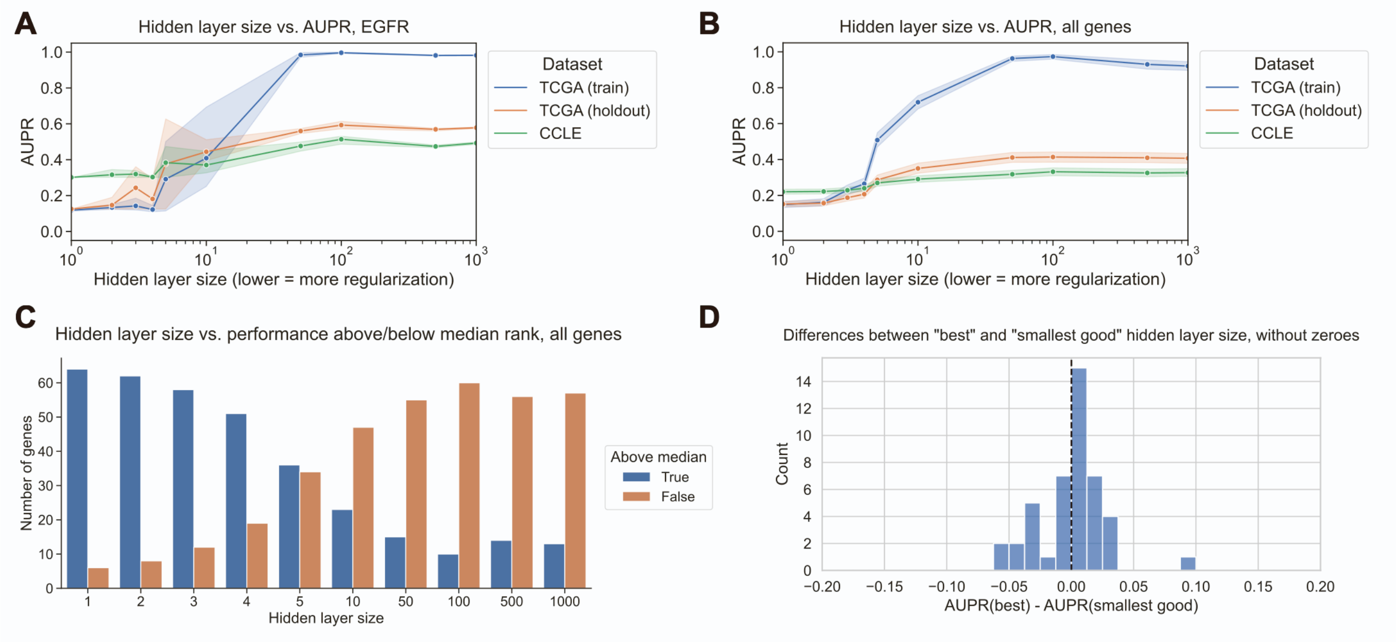

Figure S10: Summary of performance for TCGA to CCLE generalization using 5-layer fully connected neural network, analogous to results shown in Figure 5 for 3-layer network. All experiments used expression of top 8000 genes by mean absolute deviation, for computational reasons. In the "best" vs. "smallest good" analysis, 27/71 genes had better performance for the best model, and 17/71 had better performance for the smallest good model, with 26/71 genes where the best and smallest good models were equal.
